# Responsiveness of Elite Cultivars vs. Ancestral Genotypes of Barley to Beneficial Rhizosphere Microbiome, Supporting Plant Defense Against Root-Lesion Nematodes

**DOI:** 10.3389/fpls.2021.721016

**Published:** 2021-08-19

**Authors:** Ahmed Elhady, Sakineh Abbasi, Naser Safaie, Holger Heuer

**Affiliations:** ^1^Institute for Epidemiology and Pathogen Diagnostics, Julius Kühn-Institute, Federal Research Center for Cultivated Plants, Braunschweig, Germany; ^2^Department of Plant Protection, Faculty of Agriculture, Benha University, Benha, Egypt; ^3^Department of Plant Pathology, Tarbiat Modares University, Tehran, Iran

**Keywords:** plant defense, induced resistance, domestication, *Pratylenchus neglectus*, barley, nematode, rhizosphere microbiome, suppressive soil

## Abstract

Harnessing plant-microbe interactions to advance crop resistance to pathogens could be a keystone in sustainable agriculture. The breeding of crops to maximize yield in intensive agriculture might have led to the loss of traits that are necessary for beneficial plant-soil feedback. In this study, we tested whether the soil microbiome can induce a stronger plant defense against root-lesion nematodes in ancestral genotypes of barley than in elite cultivars. Plants were grown in a sterile substrate with or without the inoculation of rhizosphere microbiomes, and *Pratylenchus neglectus* was inoculated to the roots. Unexpectedly, elite cultivars profited significantly more from the microbiome than ancestral genotypes, by the reduction of nematodes in roots and the increased shoot weight relative to control plants. The elite cultivars had higher microbial densities in the rhizosphere, which were correlated with root weight. The structure of the bacterial and fungal community of elite and ancestral genotypes differed, as compared by 16S rDNA or internal transcribed spacer amplicon profiles in denaturing gradient gel electrophoresis. The elite cultivars differed in responsiveness to the microbiome. For the most responsive cultivars Beysehir and Jolgeh, the strong microbe-induced suppression of nematodes coincided with the strongest microbe-dependent increase in transcripts of salicylic acid-regulated defense genes after nematode invasion, while the jasmonate-regulated genes *LOX2* and *AOS* were downregulated in roots with the inoculated microbiome. The microbe-triggered modulation of defense gene expression differed significantly between elite and ancestral genotypes of barley. Soil microbiomes conditioned by maize roots suppressed the nematodes in elite cultivars, while the corresponding bulk soil microbiome did not. In conclusion, cultivars Beysehir and Jolgeh harbor the genetic background for a positive plant-microbiome feedback. Exploiting these traits in breeding for responsiveness to beneficial soil microbiomes, accompanied by soil biome management for compatible plant-microbe interactions, will support low-input agriculture and sustainability.

## Introduction

The domestication of crops is an ongoing multistage process that has taken place over the past 12,000 years, as plants have been adapted to human needs and agricultural systems using selection, polyploidy, and introgression (Fernie and Yan, 2019). The recent acceleration of this process together with chemical fertilizers and pesticides has enabled us to provide food for the ever-growing human population (Khush, 2001).

Microbiomes in the rhizosphere of plants are in part intimately associated with particular plant species and play a key role in plant growth and health (Mendes et al., 2011; Liu et al., 2020). Roots are surrounded by a rich diversity of microorganisms that support them in nutrient acquisition, production of growth factors, defense against pathogens, and mitigate their exposure to adverse environmental stress depending on species composition (Lugtenberg, 2015). The plant alongside its associated microbiota is considered as a unit of selection termed “holobiont” that determines the plant performance and productivity (Gopal and Gupta, 2016; Rosenberg and Zilber-Rosenberg, 2018). The composition of root exudates provided to the microbiome in the rhizosphere is determined by plant species (Badri and Vivanco, 2009; Eisenhauer et al., 2016) and can differ among genotypes of the same plant species (Micallef et al., 2009). The plant and its closely associated microbiota communicate through different channels and together modulate the structure of the microbiome in the rhizosphere (Venturi and Keel, 2016; Sasse et al., 2018; Middleton et al., 2021).

However, it has been argued that beneficial plant-microbe interactions tend to become disrupted during domestication due to trade-offs with desired plant traits, genetic costs, and relaxed selection in high-input agriculture (Porter and Sachs, 2020). Breeding can unintentionally change the structure and physiology of the rhizosphere microbiome (Pérez-Jaramillo et al., 2018; Mendes et al., 2019), so that wild and domesticated plants (Bulgarelli et al., 2015), or cultivars of the same crop (Weinert et al., 2009), differ in the structure of the rhizosphere community. The extent to which domesticated plants have lost the capacity to maintain advantageous microbiomes remains poorly understood (Hassani et al., 2019). Since the high-input agriculture is at the expense of the environment and sustainability, it has been suggested to exploit genetic traits of wild crop relatives to reinstate beneficial plant-microbe associations in elite cultivars (Pérez-Jaramillo et al., 2016). However, it is still not clear whether elite varieties profit less from rhizosphere microbiomes than their progenitors.

The evolutionary history of barley (*Hordeum vulgare* L.) is well studied (Jakob et al., 2014), and the seeds of wild relatives are available from seed banks. Root-lesion nematodes (RLN, *Pratylenchus* spp.) are endoparasitic nematodes that cause substantial yield loss in barley and many other crops worldwide, and efficient control options are lacking (Mokrini et al., 2019). Recently, we discovered that conditioning soil microbiomes by maize roots suppressed RLN in the follow-up crop by inducing systemic resistance in the plant (Elhady et al., 2018). We used this finding to generate beneficial microbiomes and investigated whether the domestication of barley affected the suppression of RLN by these microbiomes. Specifically, we tested the following hypotheses: (1) the microbiome, inoculated to roots, better supports the growth of ancestral genotypes in the presence of RLN compared with elite cultivars of barley, when growth parameters are determined relative to those of the same plant variant without inoculated microbiome; (2) the microbiome better prevents invasion of RLN in roots of ancestral genotypes than elite cultivars, when RLN are counted relative to those of the same plant variant without inoculated microbiome; (3) as elite cultivars might allocate more resources to the shoot at the expense of less root exudates for the support of microbial associations, we tested whether ancestral genotypes have higher microbial densities per root weight in their rhizosphere than elite cultivars; (4) whether the rhizosphere of ancestral genotypes and elite cultivars differs in the structure of the microbial community; (5) the microbiome-dependent regulation of defense genes in response to RLN differs between elite cultivars and ancestral genotypes. To investigate the specificity of the plant-microbiome association, we tested (6) whether the microbiome from maize rhizosphere reduces RLN in roots more pronounced than the corresponding bulk soil microbiome, in a plant genotype-dependent manner.

## Materials and Methods

### Barley Germplasm Accessions

To investigate how the domestication of barley affected its responsiveness to soil microbiota in terms of suppression of RLN invasion and plant growth during nematode attack, elite cultivars and ancestral accessions were tested. Twelve accessions with identification keys TUR-05-Bjs-HB028, HB031, HB033, HB036, HB040, HB045, HB047, HB049, HB050, HB053, HB058, and HB059 representing ancestral genotypes, *Hordeum vulgare* subsp. *spontaneum* were obtained from the National Small Grains Collection (NSGC), USDA, Aberdeen, ID, USA. The germplasm was originally collected near Mardin in Turkey (Supplementary Table 1). Two more ancestral genotypes, namely, TN374 (HS1) and S09 check/141 (HS2), were obtained from the International Center for Agriculture Research in the Dry Areas (ICARDA), Beirut, Lebanon. Elite cultivars were Igri and Valentina from Germany, Beysehir from Turkey, and Yusuf and Jolgeh from Iran.

### Nematode Inoculum

Two populations of the RLN, *Pratylenchus neglectus*, were used for all experiments. To have an axenic culture of the German population, nematodes were extracted by magnesium sulfate floatation-centrifugation of soil collected from cereal fields. Extracted *P. neglectus* were surface-sterilized by treating them on a 5-μm sieve (Cell-Trics1, Sysmex, Norderstedt, Germany) with 0.02% HgCl_2_ for 3 min, 4,000 ppm streptomycin sulfate for another 3 min, and 10 ml sterilized tap water. Nematodes were recovered in a 50-ml tube and incubated for 4 h in 5 ml 1 × CellCultureGuard (AppliChem, Darmstadt, Germany) on a rotary shaker at 150 rpm. In the end, nematodes were washed on a 5-μm sieve with sterile tap water to remove any chemicals or antibiotics. Single females were fished based on their morphological features, transferred to surface-disinfected carrot disks, incubated at 22 ± 2°C, and checked regularly under a stereomicroscope Olympus SZX12 (Olympus, Hamburg, Germany). The nematodes that successfully propagated on the carrot disks were checked by amplifying and sequencing the cytochrome c oxidase subunit I (CO1) to confirm the purity of the nematode culture (European Mediterranean Plant Protection Organization, 2016). The second population of *P. neglectus* was obtained from the Iranian Research Institute of Plant Protection (IRIPP). Nematodes were collected from the soil of a wheat field near Guilan, Iran. The nematodes were morphologically identified and multiplied on wheat plants in the greenhouse. To prepare inoculum for infection, roots of 3-month-old culture were cut into 1-cm pieces and placed on a 100-μm sieve in a mist chamber. The nematodes were collected every 3 days over 2 weeks and used directly for the infection of barley roots.

### Responsiveness of Modern and Ancestral Genotypes to Microbiomes From Maize Rhizosphere

To investigate the impact of domestication of barley genotypes on their responsiveness to soil microbiomes, two independent experiments were conducted differing in the set of ancestral and elite genotypes, RLN population, and the soil in which the maize rhizosphere microbiome was generated.

The first experiment was carried out in Germany with soil from a field near Ahlum, Germany (52°09′58.8″N 10°34′59.3″E). It was a loamy silt (clay 11.7%, silt 80.5%, and sand 7.8%), pH 6.5, C/N ratio of 9, carbon content 0.95%, total nitrogen 10%, and humus content 1.6%. The phosphor, potassium, and magnesium contents were 6.9, 16.1, and 7 mg/100 g soil, respectively. The soil was collected in November 2019 after a rotation of winter barley in 2018 and sugar beet in summer 2019. To obtain the core microbiome for barley inoculation, soils were collected from the field and air-dried for 48 h before cultivation. Maize (*Zea mays* L. cv. Colisee) was grown in 1-L pots filled with field soil to condition the soil microbiome. Pots were kept in the greenhouse at 24°C and a 16-h photoperiod for 12 weeks. Plants were watered every 2–3 days and fertilized with 1.5 g of the 3- to 4-month dose Osmocote® Pro (ICL, Nordhorn, Germany). The conditioned microbiomes were extracted from 15 g rooted soil in a Stomacher blender (Seward, London, UK) three times with 15 ml sterile saline at high speed for 60 s. Soil particles were sedimented in the vertically standing tubes for 3 min, and the microbial suspensions of the supernatant were passed through a 5-μm sieve to remove indigenous plant-parasitic nematodes that might affect root invasion and reproduction assays (Elhady et al., 2018). The microbes were pelleted by centrifugation at 4,000 × *g* for 15 min, washed with sterile water, and resuspended in sterile water for inoculation to roots of barley grown in a sterile substrate. In this first experiment, ancestral barley variants were HB028, HB031, HB033, HB036, HB040, HB045, HB047, HB049, HB050, HB053, HB058, and HB059. Elite cultivars were Igri, Valentina, and Beysehir. Seeds were sown in pots filled with 150 ml 2 × autoclaved sand supplemented with 1.5 g/L of the 3- to 4-month dose Osmocote® Pro (ICL, Nordhorn, Germany). Seeds were left for 1 week to germinate. Each pot was inoculated on the surface with 25 ml of a microbiome suspension from the maize rhizosphere. The inoculation was repeated two times more every 3 days. Controls of microbiome-free plants received sterile water instead. Pots were arranged in complete randomized design in the greenhouse at 24°C and a 16-h photoperiod (*n* = 12 per variant). Before each pot was inoculated with 1,000 *P. neglectus* (juveniles and adults) by equally distributing them into four 1.5-cm-deep holes around the shoot, plants were grown for 10 days for the establishment of the microbiome in the rhizosphere. Ten days after infection, nematode numbers in the root were determined, roots and shoots were weighed, and bacterial and fungal communities in the rhizosphere were characterized. To do this, roots with adhering soil were sampled into sterile Stomacher80 bags and extracted three times with 15 ml sterile saline in a Stomacher blender (Seward, London, UK). Supernatants were decanted into 50-ml tubes, and 0.1 ml was used for serial dilutions and spread plating on R2A agar (Merck, Germany) to determine microbial colony-forming units (CFU) 2, 3, and 7 days after incubation at 28°C. The remaining suspension was centrifuged at 4,000 × *g* for 15 min. The pellets were stored at −20°C until DNA extraction for microbiome analysis by denaturing gradient gel electrophoresis (DGGE). Roots in the Stomacher bags were washed to remove the remaining soil, dried with a paper towel, and weighed. Roots were bleached and stained with 1% acid fuchsin (Bybd et al., 1983) to count the nematodes in the roots at 20 × magnification under a stereomicroscope (Olympus SZX12).

The second experiment was carried out in Iran with soil from a field near Meshgin Shahr, Ardabil, Iran (38°24′17.0″N 47°39′54.0″E). The soil had a texture of clay 2%, silt 8%, and sand 90%, pH 7.9, electrical conductivity 2.86 dS/m, carbon 0.9%, and nitrogen 0.7%. Spring barley and winter wheat were cultivated in the field before sampling. In this experiment, ancestral barley variants HS1 and HS2 were compared with elite cultivars Yusuf and Jolgeh. The inoculation of the plants with maize rhizosphere microbiome or sterile water, then with the *P. neglectus* population from IRIPP, and sampling was performed as in the first experiment to determine the microbiome-induced change in root and shoot weights and the nematode numbers in the root.

### DGGE Analysis of Microbiomes Associated With Barley Genotypes

To compare the structure of microbial communities, 0.25 g of the microbial pellets from the rhizospheres of the elite cultivars Igri, Valentina, and Beysehir and the ancestral genotypes HB047, HB049, and HB050 (collected from experiment 1) were used to extract total community DNA using a FastPrep FP120 Bead Beating System (MP Biomedicals, Heidelberg, Germany) for 30 s at a high speed and a FastDNA SPIN Kit for Soil (MP Biomedicals), following the instructions of the manufacturer. The quality of DNA was checked by agarose gel electrophoresis. To compare microbial communities of ancestral and elite genotypes within the same DGGE gel, the three elite varieties and three of the ancestral genotypes (i.e., HB047, HB049, and HB050) were selected for the analysis. The bacterial 16S rRNA gene fragments were amplified using primers F984GC and R1378 as previously described (Heuer et al., 1997). The fungal internal transcribed spacers (ITS) were amplified in a nested PCR approach. First, primer pair ITS1F/ITS4 were used according to the study by Ihrmark et al. (2012), followed by a second amplification step using ITS1FGC/ITS2 primers (Weinert et al., 2009). The amplified bacterial or fungal products were separated by DGGE using the PhorU2 system (Ingeny, Goes, the Netherlands) as previously described (Weinert et al., 2009). The silver-stained gels were scanned with high-resolution settings (Epson 1680 Pro, Seiko Epson Corp. Suwa, Nagano, Japan). The aligned banding patterns were used to determine pairwise Pearson's correlations using the program GelCompar II (version 6.6, Applied Maths, Sint-Martens-Latem, Belgium). Significant differences of bacterial or fungal community profiles from barley rhizospheres of elite cultivars compared with ancestral genotypes were tested by permutation tests based on the similarity matrix of the community profiles as described by Kropf et al. (2004), with 10,000 permutations.

### Microbiome-Triggered Defense Gene Expression

To quantify the microbiome-dependent regulation of defense genes of barley genotypes in response to nematodes, we explored two comparisons of interest. First, we compared the microbiome effect on defense gene expression of the contrasting elite cultivars Beysehir and Valentina, because Beysehir showed microbiome-induced resistance to *P. neglectus*, while Valentina did not respond to the inoculated microbiome. Plants of both cultivars were grown either with or without inoculated microbiome and infected with *P. neglectus*, as described earlier. After inoculation of the nematodes for 1 and 3 days, six replicate pots of each treatment were sampled. Roots were immediately frozen in liquid nitrogen. Frozen roots were ground in a mortar with a pestle. The frozen root powder was transferred to 2-ml microtubes. Total RNA was extracted using the FastRNA Pro Green Kit (MP Biomedicals) following the instructions of the manufacturer. Residual traces of DNA were removed by DNase I digestion followed by DNase inactivation and removal, using a DNA-free Kit (ThermoFisher Scientific, Waltham, MA, USA). The concentration of RNA and quality was determined using a Nanodrop 2000 spectrophotometer (ThermoFisher Scientific). The RNA was reverse-transcribed using Superscript IV and an oligo(dT)_20_ primer according to the instructions of the manufacturer (ThermoFisher Scientific). The cDNA levels of the defense genes *PRX7, PR1, HSP70, PR17B, GSL6*, and *CSD1* and the reference gene *UBQ* were analyzed by quantitative real-time PCR (qRT-PCR) using primers listed in Supplementary Table 2 (Shrestha et al., 2019) in a CFX Connect Real-Time PCR Detection System (Bio-Rad, Munich, Germany), with two technical replicates of each reaction. Amplifications were performed in 20 μl reactions using Luna® Universal qPCR Master Mix (New England BioLabs, Frankfurt am Main, Germany). Thermo cycles were as follows: initial denaturation at 95°C for 2 min, 40 cycles of a denaturation step at 95°C for 30 s, an annealing step at 60°C for 30 s, an extension step at 70°C for 30 s, and 80°C for 15 s. The fluorescence was read at the 80°C steps of each cycle. Cycles of detection (C_t_) of defense genes were corrected by C_t_ of *UBQ*, and C_t_ of defense genes from microbiome-inoculated roots were related to the mean C_t_ of the defense gene from non-inoculated roots, using the –ΔΔC_t_ method (Pfaffl, 2001). Thus, –ΔΔC_t_ > 0 indicated a higher expression of the respective defense gene in microbiome-inoculated roots than in roots of control plants without inoculated microbiome during RLN invasion.

In the second experiment on microbiome-regulated defense gene expression, we compared the expression of defense genes of the elite cultivars Jolgeh and Yusuf to those of the ancestral genotypes HS1 and HS2. The experiment was carried out along the lines of the first experiment, but plants without inoculated microbiome or nematodes served as control. Four replicate roots from each treatment variant and genotype were sampled 5 days after inoculation of *P. neglectus*. RNA extraction and cDNA synthesis were performed as described earlier. For qRT-PCR of genes *EXPB1, PR1, PR5, LOX2, AOS*, and *UBQ*, primers listed in Supplementary Table 3 were used. PCR conditions and thermocycles were as described earlier. The C_t_ of defense genes were corrected by C_t_ of *UBQ*, and C_t_ of defense genes from nematode-inoculated roots with or without microbiome were related to the mean C_t_ of the defense gene from control plants, using the –ΔΔC_t_ method (Pfaffl, 2001). Thus, –ΔΔC_t_ > 0 indicated a higher expression of the respective defense gene in nematode-inoculated roots, either with or without inoculated microbiome, than in roots of control plants that were not exposed to RLN or soil microbiome. All C_t_ values are shown in Supplementary Data 4.

### Responsiveness of Different Elite Cultivars to Microbiomes From Maize Rhizosphere and Bulk Soil Regarding the Invasion of *P. neglectus*

To investigate the specificity of the plant-microbiome association, we tested whether the microbiome from maize rhizosphere reduces RLN in roots more pronounced than the corresponding bulk soil microbiome, in a plant genotype-dependent manner. The microbial suspensions of maize rhizosphere or bulk soil were extracted from 15 g rooted or bulk soil as described earlier and were inoculated to 1-week seedlings of barley cultivars Igri, Valentina, and Beysehir growing in pots filled with 150 ml autoclaved sand. A control of microbiome-free plants received only sterile water. After the establishment of the microbiome in the rhizosphere for 10 days, plants were infected with 1,000 *P. neglectus* (mixed stages) as described earlier. Ten days after infection, roots were sampled, washed, and bleached, and nematodes were microscopically counted after staining with 1% acid fuchsin.

### Statistical Analyses

The ANOVA was performed using the procedure GLIMMIX of the statistical software package SAS 9.4 (SAS Institute Inc., Cary, NC, USA) to fit generalized linear (mixed) models. Kenward–Roger's procedure was used to estimate the degrees of freedom. When comparing the effect of inoculated microbiome between elite cultivars and ancestral genotypes in the two independent experiments with various genotypes, experiment and genotype were treated as random effects. For multiple comparisons using the LSMEANS statement, the *P*-value was adjusted by Tukey's method. Multivariate ANOVA (MANOVA) on microbiome-dependent transcript levels of all tested defense genes was performed using the procedure generalized linear mixed model (GLMM). Details of statistical analyses are explained in Supplementary Data 5.

## Results

### Growth Response of Elite Cultivars and Ancestral Genotypes of Barley to the Inoculated Microbiome

**Figure 1:**
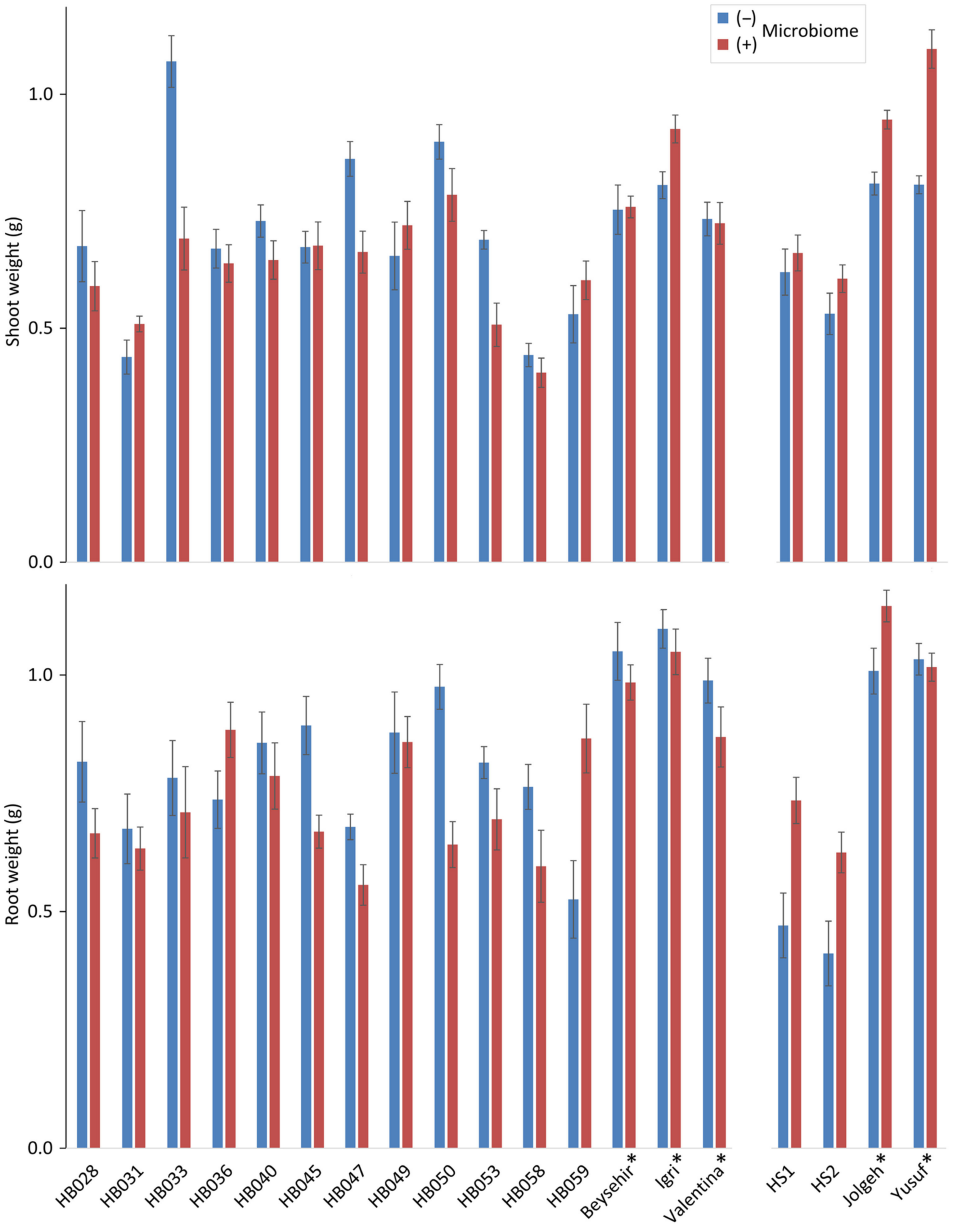
Responsiveness of elite cultivars (indicated by a star) and ancestral genotypes of barley to rhizosphere microbiomes regarding plant growth in the presence of root-lesion nematodes, *Pratylenchus neglectus*. Mean shoot and root weights from the genotypes used in the two independent experiments are separated on the horizontal axis. Error bars indicate SE (*n* = 12 plants with (+) and 12 plants without (–) inoculated microbiome per genotype of barley).

Comparing the elite barley cultivars to various ancestral genotypes of barley resulted in significant responsiveness with respect to the effect of the inoculated microbiome on growth in the presence of RLN. As expected, the shoot and root weights of elite cultivars were significantly higher compared with ancestral barley (*P-*values = 0.006 and 0.001, respectively) (Figure 1; Table 1). Overall, the inoculated microbiome had no significant effect on barley growth (*P-*value = 0.075 for shoot and *P-*value = 0.345 for root). However, it was different between elite and ancestral genotypes as indicated by the significant interaction effect on shoot weight (*P-*value = 0.0001 for microbiome^*^elite cultivar, Table 1). Especially the barley cultivars Jolgeh, Yusuf, and Igri showed better shoot growth under nematode attack with inoculated microbiome than without it (Figure 1). Cultivar Jolgeh also had exceptionally high microbiome-supported root growth. For the elite cultivars, a decreasing trend was shown in the root/shoot ratio after the addition of the microbiome (1.33 ± 0.03 compared with 1.15 ± 0.05, mean ± SE), which was not observed for the ancestral genotypes (1.10 ± 0.08 compared with 1.14 ± 0.05).

**Table 1:** Generalized linear mixed models (GLMM) for microbiome-dependent responses of elite cultivars and ancestral genotypes of barley.

	***P*** **-value of fixed effect^a^**		
**Dependent variable**	**microbiome (+/–)**	**elite_cv (1/0)**	Interaction microbiome^*^elite_cv
Shoot weight	0.0751	0.0057	0.0001
Root weight	0.3451	0.0001	0.9687
Log (root-lesion nematodes)	0.0001	0.0012	0.0001

a *Model: Dependent variable = microbiome elite_cv Microbiome*

* *elite_cv, with genotype and experiment as random effects (procedure GLIMMIX, SAS 9.4)*.

### Microbiome-Supported Defense of Elite and Ancestral Genotypes Against RLN

**Figure 2:**
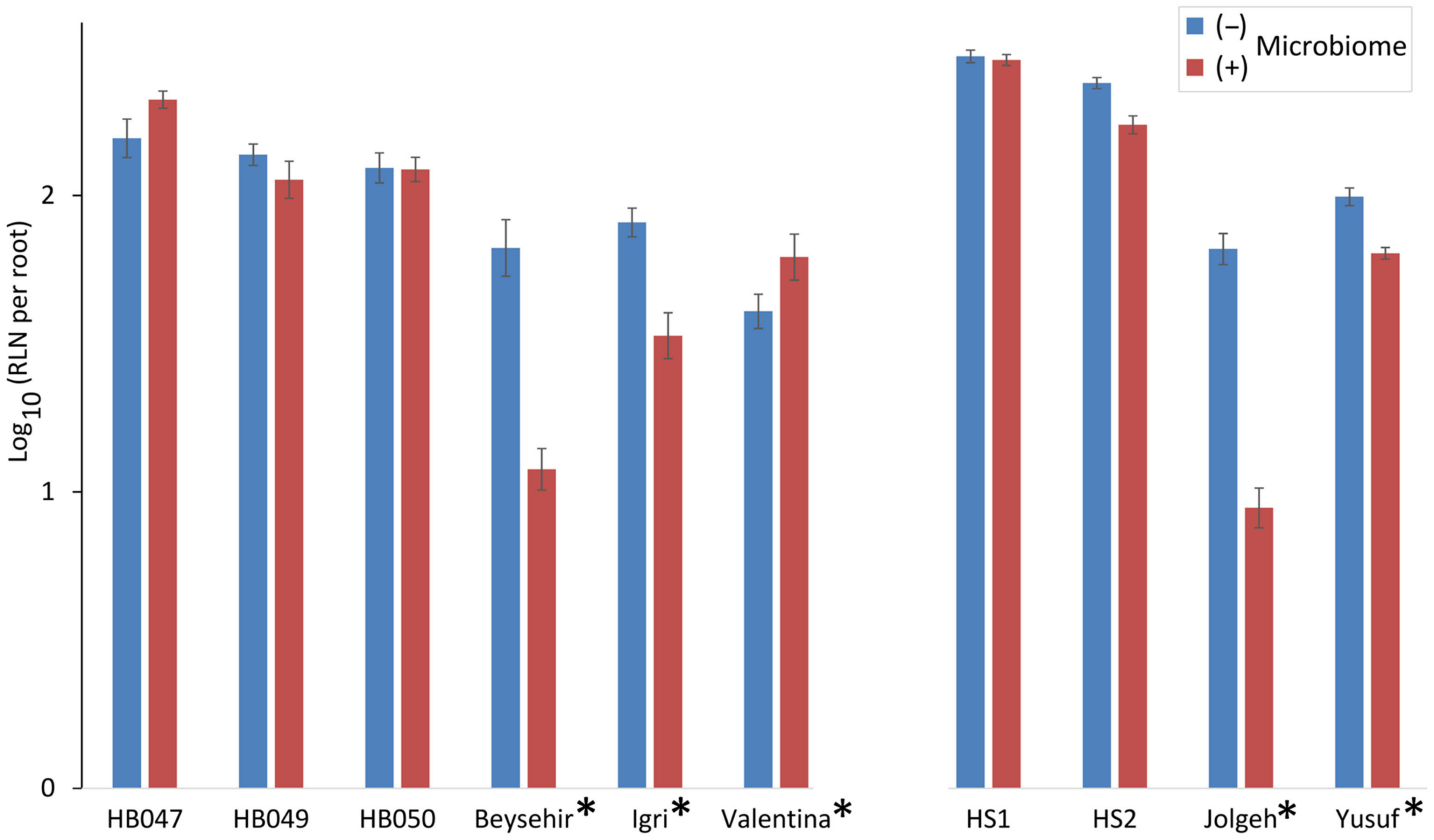
Responsiveness of elite cultivars (indicated by a star) and ancestral genotypes of barley to rhizosphere microbiomes regarding the invasion of root-lesion nematodes (RLN) within 2 weeks. Mean log-transformed numbers of RLN per root system from the two independent experiments are separated on the horizontal axis. Error bars indicate SE (*n* = 12 plants with (+) and 12 plants without (–) inoculated microbiome per genotype of barley).

The inoculated microbiome had a suppressive effect on the nematodes, which was more pronounced for elite cultivars than for ancestral genotypes in both experiments (Figure 2). This was proven by the significance of the effects of the factors microbiome (*P-*value < 0.0001) and elite cultivar (*P-*value = 0.001) as well as the significant interaction effect of the microbiome and elite cultivar (*P*-value < 0.0001) (Table 2). The rhizosphere microbiome most strongly reduced the nematode numbers in roots of cultivars Beysehir and Jolgeh by 82 or 87%, respectively. For cultivar Igri, the microbiome-induced reduction of nematodes was moderate with 59% on average. The microbiome did not reduce nematode numbers in roots of the ancestral genotypes HB047, HB050, and HS1 and the elite cultivar Valentina (Figure 2). When relating the nematode numbers to root weight, as larger roots may attract more nematodes, still the microbiome induced a much stronger reduction of nematodes in elite varieties compared with ancestral genotypes (Supplementary Figure 1).

**Table 2:** Generalized linear mixed models of microbiome-dependent defense gene expression of the barley cultivars Beysehir and Valentina, 1 and 3 days postinoculation (dpi) of root-lesion nematodes.

	***P*** **-value of fixed effect**		
**Dependent variable (–ΔΔC_**t**_)**	**cultivar**	**dpi**	Interaction cultivar*dpi
*PRX7*	0.2904	0.1942	0.5092
*PR1*	0.0002	0.0868	0.0546
*HSP70*	0.0035	0.0008	0.8373
*PR17B*	0.0001	0.5052	0.0009
*GSL6*	0.1726	0.7786	0.3548
*CSD1*	0.0040	0.2911	0.8946
MANOVA (GLMM)	0.0001	0.0001	0.0010

### Microbial Density in the Rhizosphere of Elite and Ancestral Genotypes of Barley

**Figure 3:**
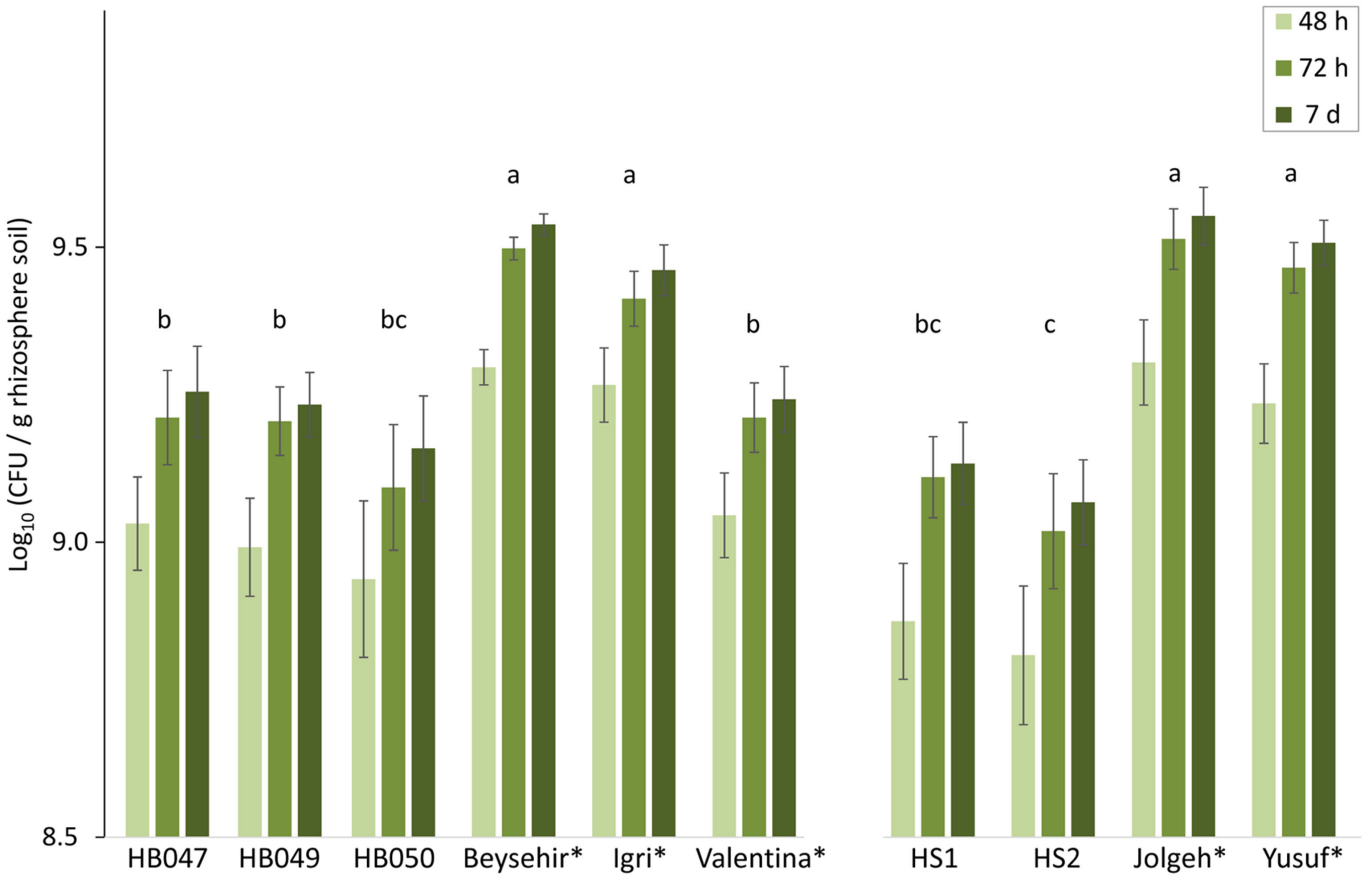
Microbial density in the rhizosphere of elite cultivars (indicated by a star) and ancestral genotypes of barley 4 weeks after inoculation of a soil microbiome to the roots. Colony-forming units (CFU) on R2A plates were counted after 2, 3, and 7 days incubation at 28°C. Mean log-transformed CFU/g from the two independent experiments are separated on the horizontal axis. Error bars indicate SE (*n* = 6 rhizosphere samples per genotype of barley; each sample was derived from two plants). The same letter above triples of bars indicates not significant differences between genotypes (generalized linear mixed model, repeated measures, and Tukey's adjustment).

As the inoculated microbiome significantly influenced barley growth and infection in a genotype-dependent matter, we compared the microbial densities in the rhizospheres 4 weeks after inoculation of the microbiome. The elite cultivars Beysehir, Igri, Jolgeh, and Yusuf harbored significantly more cultivable microbes in their rhizosphere compared with the ancestral genotypes and the elite cultivar Valentina (Figure 3). Over both experiments, elite cultivars had significantly higher plate counts (GLMM, *P*-value = 0.0018). Log(CFU) per root weight correlated with root weight (*R*^2^ = 0.75), which was higher for elite cultivars than for ancestral genotypes. Some of the microbes reached visible colony size on culture plates only after 2 or 7 days of incubation (Figure 3). This lag in microbial growth on agar media did not differ between rhizosphere microbes of elite and ancestral genotypes (GLMM, interaction elite cultivar^*^incubation time, *P*-value = 0.96).

### Structure of Bacterial and Fungal Rhizosphere Communities Analyzed by DGGE

**Figure 4:**
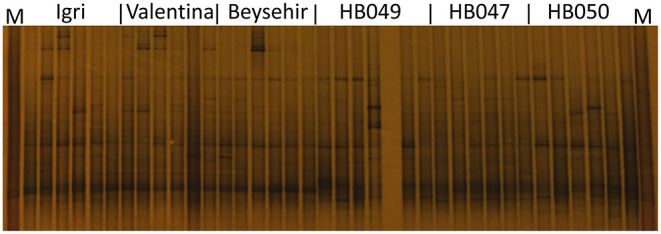
Bacterial rhizosphere communities of elite cultivars (Igri, Valentina, and Beysehir) and ancestral genotypes (HB049, HB047, and HB050) of barley, analyzed by denaturing gradient gel electrophoresis of 16S rRNA gene fragments (*n* = 6 replicate fingerprints per genotype, each replicate sample was derived from microbial extracts of roots and adhering soil of two plants). M: a marker of cloned 16S rRNA variants.

**Figure 5:**
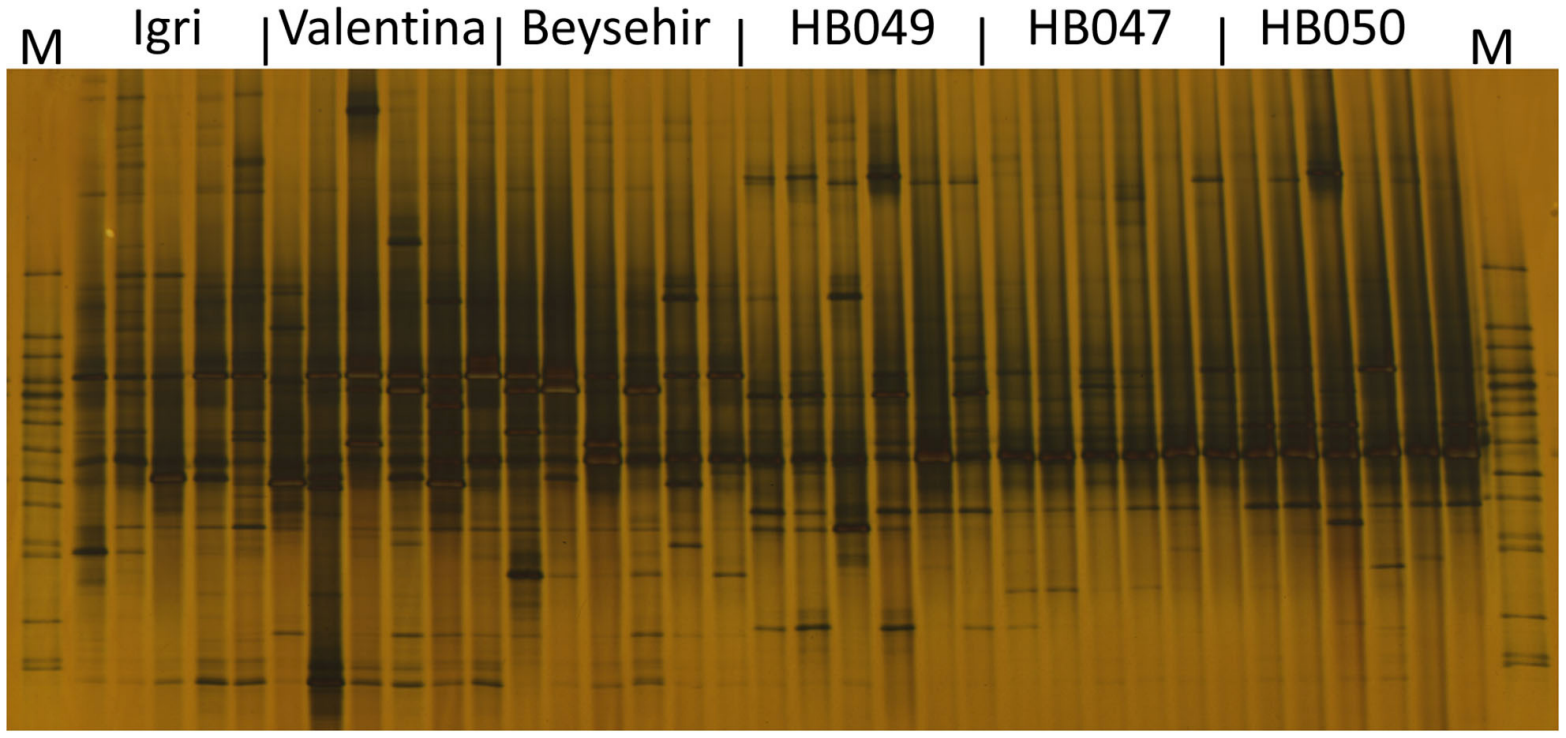
Fungal rhizosphere communities of elite cultivars (Igri, Valentina, and Beysehir) and ancestral genotypes (HB049, HB047, and HB050) of barley, analyzed by denaturing gradient gel electrophoresis of internal transcribed spacer (ITS) fragments (*n* = 6 replicate fingerprints per genotype, each replicate sample was derived from microbial extracts of roots and adhering soil of two plants). M: a marker of cloned ITS variants.

Not only the microbial density but also the structure of the community might influence the growth and defense of barley. The DGGE fingerprinting showed that most of the bands in the bacterial profiles were common to rhizospheres of all genotypes (Figure 4). However, probably more pronounced bands in the lower part of the gel (high G+C ribotypes) for ancestral barley and the upper part of the gel for elite cultivars resulted in a significant difference in the bacterial profiles between elite and ancestral genotypes (permutation test on pairwise similarities, *P*-value = 0.001). Fungal profiles showed few dominant fungal types, most of which were from the rhizosphere of elite cultivars (Figure 5). Comparison among the fungal communities of the elite and ancestral barley genotypes by a permutation test confirmed the significant difference (*P*-value = 0.001). In dendrograms, most of the bacterial or fungal profiles of the ancestral barley genotypes clustered in two groups (Supplementary Figure 2).

### Microbiome-Dependent Expression of Defense Genes in Nematode-Infested Cv. Beysehir Compared With Cv. Valentina

**Figure 6:**
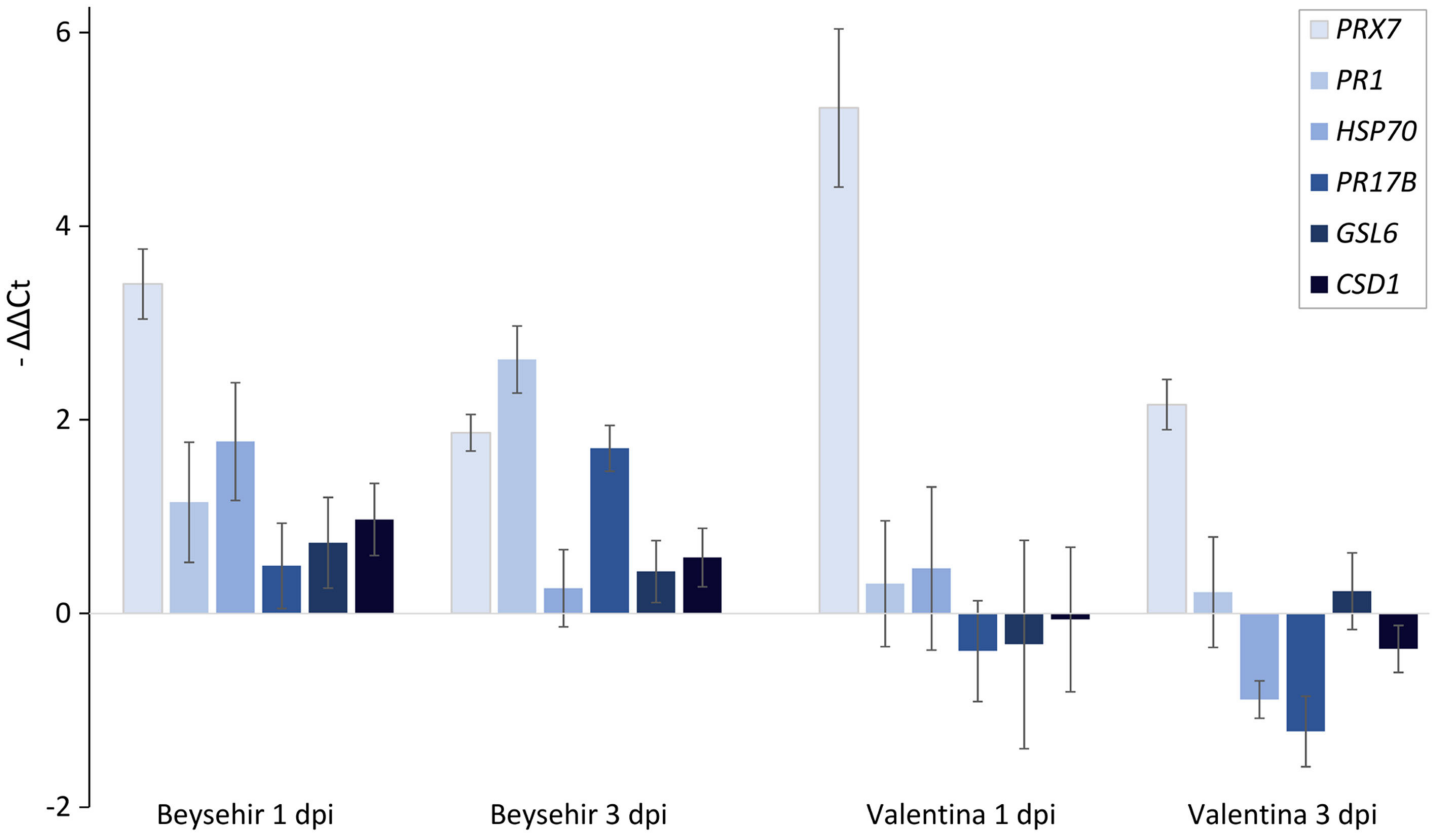
Microbiome effect on defense gene expression of the cultivar Beysehir that showed microbiome-induced resistance to *P. neglectus*, and the cultivar Valentina that did not respond to the microbiome (Figure 3), 1 day and 3 days after inoculation of *P. neglectus*. RNA was extracted, reverse-transcribed, and quantified in real-time PCR. Transcript levels were determined for roots with the inoculated microbiome, relative to expression in noninoculated roots, both normalized to the expression of the control gene *UBQ* according to the –ΔΔC_t_ method. Error bars represent SE (*n* = 6).

Among the elite cultivars tested, Beysehir and Valentina responded antithetic to the inoculated microbiome by either decreased or increased invasion of RLN, respectively. We hypothesized that this corresponds to differences in microbe-induced defense gene expression. All of the six tested defense genes of Beysehir were upregulated in microbiome-inoculated roots compared with non-inoculated roots, 1 and 3 days postinoculation (dpi) of RLN (Figure 6). In contrast, five of these genes of Valentina were either less upregulated than in Beysehir or even downregulated compared with plants without inoculated microbiome. The MANOVA revealed significant effects of the cultivar and the dpi (Table 3). Overall genes, microbe-induced expression as measured by –ΔΔC_t_ decreased from 1 to 3 dpi in Beysehir by 12%, but in Valentina by even 97% (significant interaction effect cultivar^*^dpi, MANOVA, Table 3). Genes *PR1* and *PR17B* increased in microbe-induced transcript levels from 1 to 3 dpi in Beysehir but decreased in Valentina. Both genes and *CSD1* had significantly higher microbe-induced expression levels in Beysehir than in Valentina. In summary, contrasting microbe-induced defense gene expression in response to ongoing root invasion by RLN corresponded well with contrasting microbe-induced suppression of the nematodes by cultivars Beysehir and Valentina.

**Table 3:** Generalized linear mixed models of microbiome-dependent defense gene expression of elite cultivars (Jolgeh and Yusuf) and ancestral genotypes (HS1 and HS2) of barley, 5 days after inoculation of RLN.

	***P-*** **value of fixed effect^a^**		
**Dependent variable (–ΔΔC_**t**_)**	**microbiome (+/–)**	**elite_cv (1/0)**	Interaction microbiome^*^elite_cv
*EXPB1*	0.0001	0.013	0.0001
*PR1*	0.0001	0.016	0.1970
*PR5*	0.0001	0.267	0.0100
*LOX2*	0.0001	0.135	0.0003
*AOS*	0.0006	0.044	0.7850
MANOVA (GLMM)	0.0001	0.0001	0.0001

a *Model: –ΔΔC_t_ = microbiome elite_cv microbiome*

* *elite_cv; random effect: genotype*.

### Microbiome-Dependent Regulation of Defense Genes in Response to Nematodes in Elite Cultivars Compared With Ancestral Genotypes of Barley

**Figure 7:**
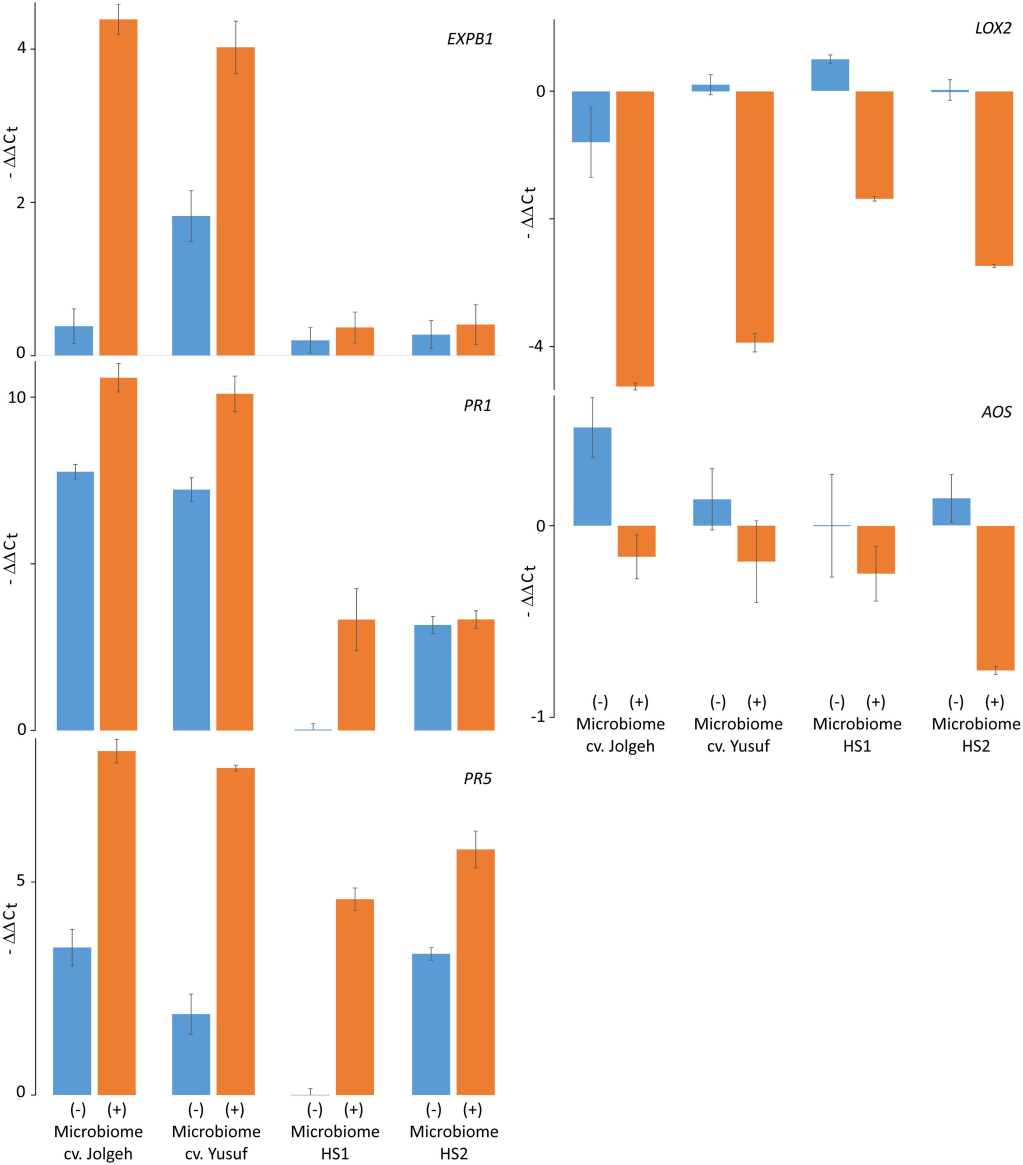
Microbiome effect on defense gene response to RLN (*P. neglectus*) infestation in roots of elite cultivars (Jolgeh and Yusuf) and ancestral genotypes (HS1 and HS2) of barley. RNA was extracted, reverse-transcribed, and quantified in real-time PCR. The expression of genes *EXPB1, PR1, PR5, LOX2*, and *AOS* 5 days after inoculation of RLN was determined relative to expression in noninfected roots and normalized to gene expression of the control gene *UBQ* according to the –ΔΔC_t_ method. Error bars represent SE (*n* = 4).

The microbe-induced expression of salicylic acid (SA)- and jasmonate (JA)-regulated defense genes of the elite cultivars Jolgeh and Yusuf differed in comparison with the ancestral genotypes HS1 and HS2. Overall five defense genes, the MANOVA revealed a significant effect of the microbiome inoculation on gene expression and the significant differences of the elite cultivars compared with the ancestral genotypes in transcript levels (Table 3). Five days after inoculation of RLN, the SA-regulated genes *EXPB1, PR1*, and *PR5* were upregulated in roots of all plants with the added microbiome, compared with plants without nematode infestation, while the JA-regulated genes *LOX2* and *AOS* were downregulated (Figure 7). The upregulation of SA-regulated genes and the downregulation of JA-regulated, respectively, were more pronounced in plants with than without inoculated microbiome. This microbiome effect was more pronounced in the elite cultivars compared with the ancestral genotypes, especially for *EXPB1, PR5*, and *LOX2* (MANOVA, interaction microbiome^*^elite_cv, Table 3). *EXPB1* was hardly influenced by the microbiome in ancestral HS1 and HS2 but drastically changed expression in elite cultivars Jolgeh and Yusuf (Figure 7). For *PR1* and *PR5*, the genotypes HS1 and HS2 reacted differently to the microbiome, while it enhanced defense in both elite cultivars. *LOX2* was slightly upregulated in HS1, HS2, and Yusuf without inoculated microbiome, while it was downregulated in plants with the inoculated microbiome, which was much more pronounced in elite compared with ancestral genotypes. *AOS* was upregulated in all variants without the inoculated microbiome, especially in cv. Jolgeh, while *AOS* was downregulated compared with non-infested controls in roots with inoculated microbiome (Figure 7). In summary, the inoculation of the microbiome to roots of barley changed the expression of defense genes more severely in elite cultivars than in ancestral genotypes, which is congruent with the observed microbiome-induced suppression of RLN in these barley variants (Figure 3).

### Responsiveness of Elite Cultivars to Microbiomes From Maize Rhizosphere Compared With Bulk Soil Regarding the Invasion of RLN

**Figure 8:**
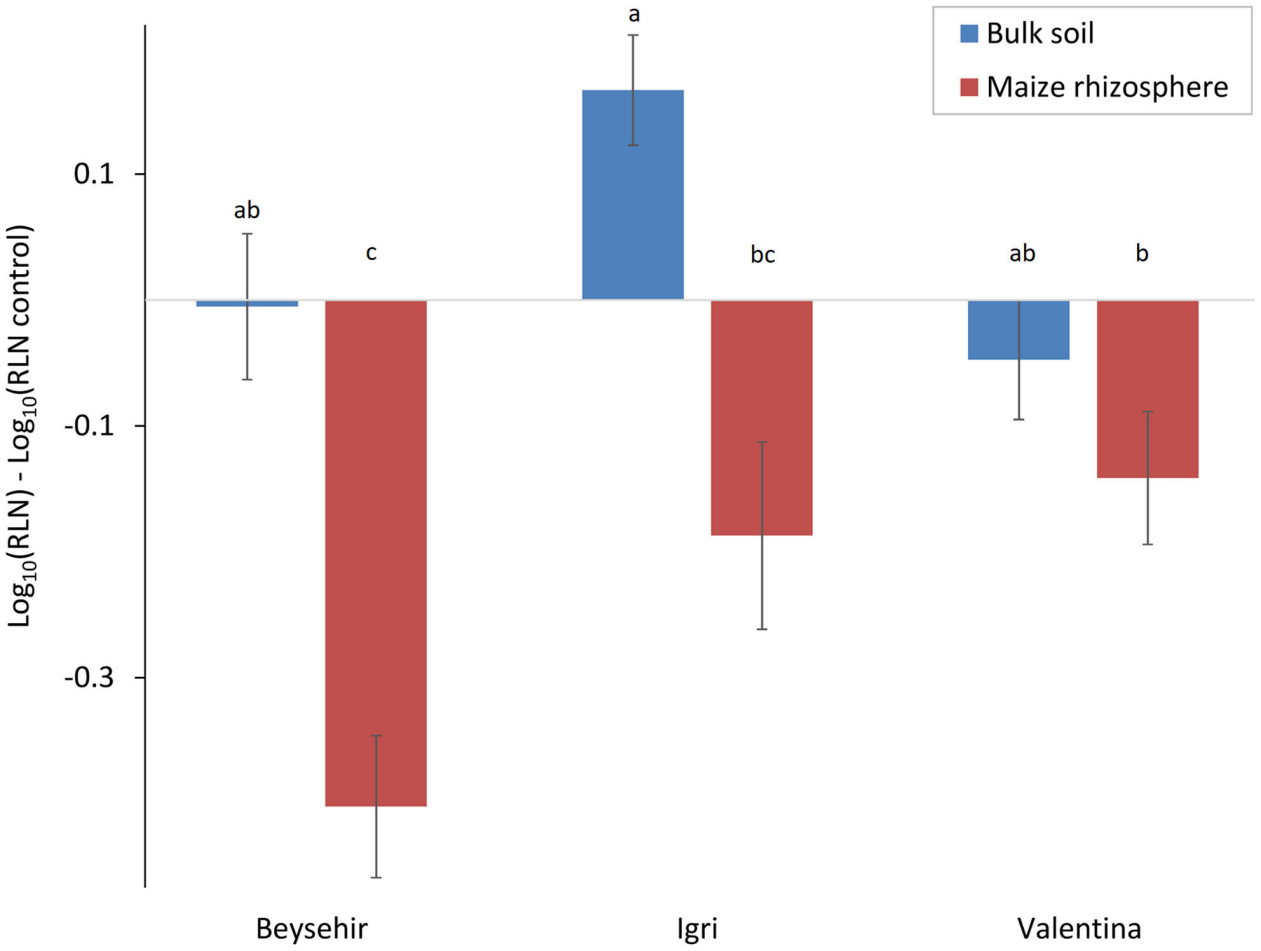
Responsiveness of elite cultivars of barley to maize rhizosphere or bulk soil microbiome regarding the invasion of RLN (*P. neglectus*), compared with a control without added microbiome. Error bars indicate SE (*n* = 12). The same letter above bars indicates no significant difference (GLMM and Tukey's adjustment).

In the previous experiments, the defense of some of the barley accessions against *P. neglectus* was triggered by a soil microbiome that was conditioned by maize roots. In this study, we tested whether the bulk soil microbiome that was not conditioned by maize roots triggers plant defense to the same extent. The cultivar and the type of inoculated microbiome significantly affected the reduction of the nematode in roots compared with the control plants without inoculated microbiome (Figure 8, GLMM, *P*-value = 0.0042 for genotype, *P*-value = 0.0001 for microbiome type). The maize microbiome significantly reduced nematode invasion into roots of cv. Beysehir, similar to the previous experiment. However, the bulk soil microbiome did not cause a significant reduction of the nematodes in the roots of cv. Beysehir. The reduction of the nematode by the maize microbiome was also observed for cv. Igri and Valentina but significantly less in Valentina compared with Beysehir, while the reduction of the nematode in Igri was intermediate (Figure 8). In contrast, the bulk soil microbiome did not significantly reduce nematode numbers in roots of cv. Valentina, and it even supported nematode invasion into roots of cv. Igri (Figure 8). These contrasting cultivar-dependent effects of the two microbiomes were reflected in a significant interaction effect of cultivar^*^microbiome (GLMM, *P*-value = 0.019). In summary, the interaction of both the plant genotype and the microbiome in the soil where the plants grow affected the number of RLN in the root, and both should be a combined target for pest control.

## Discussion

Plant defense against pathogens typically comes with the cost of reduced growth, but plants can ameliorate the cost of resistance by maintaining a protective microbiome in the rhizosphere (Karasov et al., 2017). Domestication and breeding of crops changed the microbial communities in their rhizospheres (Pérez-Jaramillo et al., 2016; Pieterse et al., 2016; Martín-Robles et al., 2020). Wild barley genotypes allocated more resources than elite cultivars to root growth compared with stem growth (Alegria Terrazas et al., 2020), which may alter root exudates and consequently the assembly of microbial communities in the rhizosphere. In this study, the potential of ancestral barley to provide genetic traits that could ameliorate plant-microbe interactions was investigated. Bacterial communities in the rhizospheres of wild and elite accessions of barley were shown to differ, which likely reflected differential evolutionary adaptations of elite cultivars to agroecosystems with relaxed biotic and abiotic stress (Bulgarelli et al., 2015; Alegria Terrazas et al., 2020). Many studies reinforced the finding of distinct microbial communities associated with wild and domesticated plants of other species (Pérez-Jaramillo et al., 2018), such as rice (Chang et al., 2021) and maize (Brisson et al., 2019). A study on tomato plants indicated that domesticated tomatoes boost more microbial-associated negative feedbacks than their wild ancestors (Carrillo et al., 2019), giving evidence that domestication might weaken a positive relationship of roots and soil microbes. Less resource allocation of bred crops to the root and maintenance of microbial communities in the rhizosphere, as well as reduced selective pressure in managed agricultural systems, may have resulted in decreased protection by the microbiome. In this study, we tested whether a protective microbiome, inoculated to roots, better supported the defense and growth of ancestral genotypes in the presence of RLN compared with elite cultivars of barley. Unexpectedly, this hypothesis was not supported by the data. Elite cultivars instead profited significantly more from the inoculated microbiome than ancestral genotypes. In contrast to the findings, Munkager et al. (2021) reported a 12% reduction in biomass of barley plants by microbiome inoculation, albeit in the absence of pathogen pressure. In this study, the microbiome effect on the reduction of invaded RLN in roots and on the increase of the shoot weight was significantly more pronounced for the elite barley cultivars compared with ancestral accessions. All plants received the same microbiome, but roots of elite and ancestral accessions shaped the microbiome in a significantly different way during early growth, as compared by 16S rDNA or ITS amplicon profiles in DGGE. Elite varieties had higher root weight and higher microbial densities in their rhizosphere, which may have resulted in higher availability of exudates in the rhizosphere and may explain a stronger enrichment of specific plant-associated bacteria and fungi. The composition of root exudates, which is under plant genetic control, was shown to shape the assembly of plant-specific rhizosphere microbiota (Pascale et al., 2019; Vieira et al., 2020). In this study, root weight was correlated with log(CFU/g), so a linear increase of roots due to breeding resulted in a strong increase of root-supported microbes. For the young barley plants used in the assays, the root/shoot ratio did not differ between elite and ancestral accessions and was much in support of the root, while mature plants of bred barley are selected to have high shoot yield and consequently low root/shoot ratios. Thus, we rejected the hypothesis that ancestral genotypes have higher microbial densities per root weight in their rhizosphere than elite cultivars, at least for young plants that are less tolerant to RLN than mature plants. The different structure of the rhizosphere microbiomes of ancestral genotypes of barley compared with those of elite cultivars did not result in enhanced protection from RLN, but it turned out that the domesticated plants profited more from the inoculated microbiomes. We used soil microbiomes as inoculants, which were conditioned by maize roots. Such conditioned microbiomes have recently been shown to suppress RLN by inducing systemic resistance in the plant (Elhady et al., 2018). The corresponding non-conditioned bulk soil microbiome did not induce the suppression of RLN in this study. Maize was shown to enrich specific beneficial plant-associated microbes in the rhizosphere, which was partly explained by root exuded benzoxazinoids or their breakdown products (Hu et al., 2018; Kudjordjie et al., 2019).

The microbiome-dependent regulation of defense genes in response to RLN was confirmed to differ between elite cultivars and ancestral genotypes of barley. Elite cultivars showed higher responsiveness to the inoculated microbiome in the upregulation of SA-dependent defense genes on RLN invasion. For the most responsive cultivars Beysehir and Jolgeh, the stronger microbe-induced suppression of nematodes coincided with the strongest microbe-dependent increase in the transcripts of SA-regulated defense genes after nematode invasion, while the JA-regulated genes *LOX2* and *AOS* were downregulated in roots with the inoculated microbiome. This corresponded to the antagonistic cross-talk among SA defense (*PR5*) and JA signaling pathways (*AOS*) (Aerts et al., 2021). Based on the studies of plant interactions with microbiota, plant resistance to pathogens is partially modulated by the symbiosis of plants with specific groups of microbes, recognized as microbial-induced systemic resistance (Enebe and Babalola, 2019). It was shown that specific rhizosphere bacteria attach to the cuticle of plant-parasitic nematodes in the rhizosphere and induce plant defense upon root invasion of the nematode (Topalović et al., 2020; Elhady et al., 2021). This is a putative mechanistic link between the structure of the rhizosphere microbiome and the fast induction of plant defense. Another mechanistic link might be the defense priming of the plant by signaling compounds from the rhizosphere microbiome, as recently shown for soybean plants attacked by RLN (Adss et al., 2021).

Not all tested elite cultivars showed equally good responsiveness to the inoculated microbiome. Partial resistance against RLN was occasionally reported, but the microbiome has not been considered to play a role in resistance (Galal et al., 2014). As QTL expression might depend on the induction by a beneficial microbiome, the resistance phenotype might not be reproducible without controlling for the microbiome in the rhizosphere. In contrast to cv. Beysehir, cv. Valentina responded to the inoculated microbiome by the increased invasion of RLN, which corresponded to a significantly different microbe-induced defense gene expression. Five of the six defense genes tested were either less upregulated in cv. Valentina than in cv. Beysehir or even downregulated compared with plants without inoculated microbiome. Genes *PR1* and *PR17B* increased in microbe-induced transcript levels in Beysehir from 1 to 3 days after inoculation of RLN but decreased in Valentina, suggesting accumulating defense suppression by RLN in roots of cv. Valentina. Plants can alleviate costs associated with the defense of pathogens by the strict regulation of gene expression, which still has a trade-off with growth (Hu et al., 2018; Wang et al., 2021), or they may recruit protection from other species (Heil, 2002; Heil and Baldwin, 2002). Recent studies on the root microbiome of barley focused on selecting genotypes with the optimized accommodation of beneficial microbiota that may help to mitigate costs in defense against pathogens (Zuccaro and Langen, 2020).

While the ancestral genotypes of barley can provide the genetic resources to cope with environmental stress (Fernie and Yan, 2019; Cope et al., 2020; Kreszies et al., 2020), the genotypes of cultivars Beysehir and Jolgeh seem to harbor a genetic background that encourages pronounced positive plant -microbiome feedback, supporting the regulation of plant defense against RLN. The responsiveness of plant genotypes to associated microbiomes can reduce or increase defense costs (Karasov et al., 2017; Wang et al., 2020). In this study, we showed how the responsiveness of plants to a beneficial soil microbiome can be tested for diverse barley genotypes. Exploiting these traits in breeding for responsiveness to beneficial soil microbiomes (Zuccaro and Langen, 2020), accompanied by soil biome management for compatible plant-microbe interactions (Bell et al., 2019), will support low-input agriculture and sustainability.

## Data Availability Statement

The original contributions presented in the study are included in the article/Supplementary Materials, further inquiries can be directed to the corresponding authors.

## Author Contributions

AE and HH conceived the research idea, conducted a literature search, designed the experiments, hosted SA, and supervised the work. AE acquired the funding. SA acquired a travel grant for her research visit to the Julius Kühn-Institute. AE and SA performed the main experiment at the Julius Kühn-Institute. AE performed the gene expression analysis of Beysehir vs. Valentina. SA and NS repeated the first experiment with other genotypes. HH and AE wrote and assembled the manuscript. All authors contributed to writing, reviewing, and approval of the final manuscript for submission.

## Conflict of Interest

The authors declare that the research was conducted in the absence of any commercial or financial relationships that could be construed as a potential conflict of interest.

## Publisher's Note

All claims expressed in this article are solely those of the authors and do not necessarily represent those of their affiliated organizations, or those of the publisher, the editors and the reviewers. Any product that may be evaluated in this article, or claim that may be made by its manufacturer, is not guaranteed or endorsed by the publisher.
