# Supplementary material for: Responsiveness of Elite Cultivars vs. Ancestral Genotypes of Barley to Beneficial Rhizosphere Microbiome, Supporting Plant Defense Against Root-Lesion Nematodes
Source: Front Plant Sci. 2021 Aug 19;12:721016. doi: 10.3389/fpls.2021.721016 (PMC8418270; doi:10.3389/fpls.2021.721016)
Supplement: Supplementary file 1 [file Data_Sheet_1.docx]

Supplementary Material

Responsiveness of elite cultivars vs. ancestral genotypes of barley to beneficial rhizosphere microbiome, supporting plant defense against root-lesion nematodes

**Ahmed Elhady^1,2*^, Sakineh Abbasi^1,3^, Naser Safaie^3^, Holger Heuer^1^**

^1^Institute for Epidemiology and Pathogen Diagnostics, Julius Kühn-Institute, Federal Research Centre for Cultivated Plants, Braunschweig, Germany

^2^Department of Plant Protection, Faculty of Agriculture, Benha University, Benha, Egypt

^3^Department of Plant Pathology, Tarbiat Modares University, Tehran, Iran

* Correspondence:
Ahmed Elhady
ahmed.gomaa@julius-kuehn.de

**Supplementary Table S1.** Wild and domesticated genotypes of barley used in this study.

| Inventory | Taxon | Plant ID | Type | Country |
| --- | --- | --- | --- | --- |
| PI 662166 HO11ID SD | *Hordeum vulgare* subsp. *spontaneum* | TUR-05-Bjs-HB-059 | Wild type | Turkey |
| PI 662165 HO11ID SD | *Hordeum vulgare* subsp. *spontaneum* | TUR-05-Bjs-HB-058 | Wild type | Turkey |
| PI 662163 HO11ID SD | *Hordeum vulgare* subsp. *spontaneum* | TUR-05-Bjs-HB-053 | Wild type | Turkey |
| PI 662162 HO11ID SD | *Hordeum vulgare* subsp. *spontaneum* | TUR-05-Bjs-HB-050 | Wild type | Turkey |
| PI 662161 HO11ID SD | *Hordeum vulgare* subsp. *spontaneum* | TUR-05-Bjs-HB-049 | Wild type | Turkey |
| PI 662159 HO11ID SD | *Hordeum vulgare* subsp. *spontaneum* | TUR-05-Bjs-HB-047 | Wild type | Turkey |
| PI 662158 HO11ID SD | *Hordeum vulgare* subsp. *spontaneum* | TUR-05-Bjs-HB-045 | Wild type | Turkey |
| PI 662157 HO11ID SD | *Hordeum vulgare* subsp. *spontaneum* | TUR-05-Bjs-HB-040 | Wild type | Turkey |
| PI 662156 HO11ID SD | *Hordeum vulgare* subsp. *spontaneum* | TUR-05-Bjs-HB-036 | Wild type | Turkey |
| PI 662154 HO11ID SD | *Hordeum vulgare* subsp. *spontaneum* | TUR-05-Bjs-HB-033 | Wild type | Turkey |
| PI 662152 HO11ID SD | *Hordeum vulgare* subsp. *spontaneum* | TUR-05-Bjs-HB-031 | Wild type | Turkey |
| PI 662150 HO11ID SD | *Hordeum vulgare* subsp. *spontaneum* | TUR-05-Bjs-HB-028 | Wild type | Turkey |
| HS1 | *Hordeum vulgare* subsp. *spontaneum* | TN374 | Wild ecotype | Iran |
| HS2 | *Hordeum vulgare* subsp. *spontaneum* | S09 check/141 | Wild ecotype | Iran |
| Cv. Igri | *Hordeum vulgare* L. | Winter barley | Domesticated | Germany |
| Cv. Beysehir | *Hordeum vulgare* L. | Winter barley | Domesticated | Turkey |
| Cv. Valentina | *Hordeum vulgare* L. | Winter barley | Domesticated | Germany |
| Cv. Jolgeh | *Hordeum vulgare* L. |  | Domesticated | Iran |
| Cv. Yusof | *Hordeum vulgare* L. |  | Domesticated | Iran |

**Supplementary Table S2.** Primers used in this study for quantification of barley defense gene expression by quantitative real time PCR in the comparison of microbiome effects on cv. Beysehir and cv. Valentina. Annealing temperatur: 60°C.

| Gene | Sequence 5’ – 3’ |
| --- | --- |
| *HvUBQ* | F: CAGTAGTGGCGGTCGAAGTG, R: ACCCTCGCCGACTACAACAT |
| *HvPR1* | F: GGACTACGACTACGGCTCCA, R: GGCTCGTAGTTGCAGGTGAT |
| *HvPR17b* | F: CGAGGTTCCTCGACTACTGC, R: ATCACATTCAGCCTCCGAAC |
| *HvCSD1* | F: ACCACAGATCTTGGCACTTGAAGG, R: GACAGAACTGAACTGTTCCAGTCACG |
| *HvHsp70* | F: CCAAGAAGTCGCAGGTTTTC, R: GGAATGCCAGAAAGGTCAAA |
| *HvPrx7* | F: TACCTCTCACATGTCAGCGGC, R: TACTACTTCGACCTGATCGCG |

Shrestha, A., Elhady, A., Adss, S., Wehner, G., Böttcher, C., Heuer, H., et al. (2019). Genetic differences in barley govern the responsiveness to *N*-acyl homoserine lactone. *Phytobiomes J.* 3, 191–202. doi: 10.1094/PBIOMES-03-19-0015-R

**Supplementary Table S3.** Primers designed in this study for quantification of barley defense gene expression by quantitative real time PCR, used in the comparison of genotypes HS1, HS2, cv. Yusuf, and cv. Jolgeh.

| Gene | Amplicon size | T_m_ | Sequence |
| --- | --- | --- | --- |
| *HvEXP1* | 107 | 62 | F: GTTGACCACAGTGCAAAGTATG  R: CGTGGGAGCGAATACGTTTA |
| *HvPR1* | 103 | 62 | F: CGTCTTCATCACCTGCAACTA  R: GTGCATGAGATTAGGGACGAG |
| *HvPR5* | 147 | 62 | F: AACCTTGCCATGGACTTCTC  R: GCAGAAGGTGATCTGGTAGTTATT |
| *HvLOX2* | 127 | 62 | F: CAACAACCCGGAGAACAAGA  R: GGGCAACAATCAAATGGAGATG |
| *HvAOS* | 118 | 62 | F: TCGGTGACAAAGGGATCAAC  R: CACATGCATGACTGACTCGTA |
| *Ubiquitin (HvUBQ)* | 144 | 61 | F: CCTACCCTGTGGTGCTAAAT  R: CGGCGTATGTGAAATGACTTTAT |

**Supplementary data S4.** Cycle thresholds C_t_ from quantification of gene expression by RT-qPCR.

| Genotype / Variety | Inoculation microbiome | Inoculation *P. neglectus* | C_t_ *UBQ* | C_t_ *EXPB1* | C_t_ *PR1* | C_t_ *PR5* | C_t_ *LOX2* | C_t_ *AOS* |
| --- | --- | --- | --- | --- | --- | --- | --- | --- |
| Jolgeh | 0 | 0 | 23.4 | 29.5 | 35.0 | 34.7 | 30.0 | 35.0 |
| Jolgeh | 0 | 0 | 23.3 | 29.3 | 34.0 | 35.0 | 31.1 | 34.2 |
| Jolgeh | 0 | 0 | 23.3 | 28.5 | 33.9 | 35.0 | 30.1 | 35.0 |
| Jolgeh | 0 | 0 | 23.2 | 28.3 | 34.8 | 34.6 | 30.7 | 35.0 |
| Jolgeh | 0 | 1 | 23.5 | 29.2 | 26.7 | 30.5 | 31.0 | 35.0 |
| Jolgeh | 0 | 1 | 23.4 | 29.0 | 27.1 | 31.0 | 31.3 | 34.2 |
| Jolgeh | 0 | 1 | 23.3 | 28.0 | 26.0 | 31.7 | 30.0 | 34.0 |
| Jolgeh | 0 | 1 | 23.2 | 28.0 | 27.0 | 32.5 | 32.9 | 34.1 |
| Jolgeh | 1 | 1 | 23.1 | 24.8 | 22.5 | 25.9 | 35.0 | 35.0 |
| Jolgeh | 1 | 1 | 23.4 | 24.0 | 23.9 | 26.5 | 35.0 | 35.0 |
| Jolgeh | 1 | 1 | 23.2 | 24.5 | 25.0 | 27.0 | 35.0 | 34.5 |
| Jolgeh | 1 | 1 | 23.2 | 24.4 | 23.6 | 27.4 | 35.0 | 35.0 |
| Yusuf | 0 | 0 | 23.1 | 29.3 | 35.0 | 32.8 | 29.5 | 35.0 |
| Yusuf | 0 | 0 | 23.2 | 28.9 | 33.3 | 34.0 | 30.3 | 34.2 |
| Yusuf | 0 | 0 | 23.2 | 29.0 | 34.8 | 34.0 | 30.8 | 35.0 |
| Yusuf | 0 | 0 | 23.3 | 28.4 | 35.0 | 33.6 | 30.9 | 35.0 |
| Yusuf | 0 | 1 | 23.3 | 28.0 | 27.4 | 31.5 | 30.0 | 35.0 |
| Yusuf | 0 | 1 | 23.1 | 27.1 | 28.1 | 31.9 | 30.5 | 34.7 |
| Yusuf | 0 | 1 | 23.1 | 27.1 | 26.1 | 32.9 | 30.5 | 34.0 |
| Yusuf | 0 | 1 | 23.2 | 26.0 | 27.4 | 30.4 | 30.0 | 34.8 |
| Yusuf | 1 | 1 | 23.2 | 24.0 | 24.2 | 25.8 | 34.1 | 35.0 |
| Yusuf | 1 | 1 | 23.3 | 25.9 | 23.1 | 26.2 | 34.3 | 35.0 |
| Yusuf | 1 | 1 | 23.1 | 24.4 | 24.3 | 25.9 | 34.7 | 35.5 |
| Yusuf | 1 | 1 | 23.1 | 25.1 | 25.9 | 25.7 | 34.0 | 34.3 |
| HS1 | 0 | 0 | 23.2 | 30.8 | 30.0 | 33.5 | 32.5 | 35.0 |
| HS1 | 0 | 0 | 23.4 | 31.0 | 30.0 | 34.0 | 34.0 | 34.0 |
| HS1 | 0 | 0 | 23.5 | 31.0 | 31.0 | 33.5 | 34.4 | 34.0 |
| HS1 | 0 | 0 | 23.4 | 30.4 | 30.2 | 33.8 | 32.3 | 35.0 |
| HS1 | 0 | 1 | 23.2 | 30.0 | 30.0 | 33.0 | 32.5 | 35.0 |
| HS1 | 0 | 1 | 23.4 | 31.0 | 30.1 | 34.0 | 33.0 | 34.0 |
| HS1 | 0 | 1 | 23.5 | 31.0 | 31.0 | 34.0 | 33.0 | 35.0 |
| HS1 | 0 | 1 | 23.4 | 30.4 | 30.0 | 33.8 | 32.7 | 34.0 |
| HS1 | 1 | 1 | 23.5 | 30.4 | 25.3 | 30.1 | 35.0 | 34.5 |
| HS1 | 1 | 1 | 23.4 | 30.0 | 26.9 | 29.0 | 35.0 | 34.6 |
| HS1 | 1 | 1 | 23.3 | 31.0 | 30.0 | 28.8 | 35.0 | 35.0 |
| HS1 | 1 | 1 | 23.4 | 30.5 | 25.9 | 28.6 | 35.0 | 35.0 |
| HS2 | 0 | 0 | 23.1 | 31.0 | 30.0 | 34.0 | 31.9 | 34.0 |
| HS2 | 0 | 0 | 23.1 | 31.0 | 29.9 | 34.0 | 32.0 | 34.2 |
| HS2 | 0 | 0 | 23.3 | 31.0 | 30.9 | 33.6 | 33.0 | 34.9 |
| HS2 | 0 | 0 | 23.4 | 30.6 | 29.9 | 33.0 | 32.4 | 34.2 |
| HS2 | 0 | 1 | 23.1 | 30.0 | 27.3 | 29.9 | 32.5 | 34.1 |
| HS2 | 0 | 1 | 23.2 | 31.0 | 26.2 | 30.0 | 32.0 | 34.0 |
| HS2 | 0 | 1 | 23.3 | 31.0 | 27.6 | 30.6 | 32.0 | 33.9 |
| HS2 | 0 | 1 | 23.4 | 30.6 | 27.0 | 30.9 | 32.8 | 34.7 |
| HS2 | 1 | 1 | 23.2 | 30.9 | 27.4 | 29.0 | 35.0 | 35.0 |
| HS2 | 1 | 1 | 23.2 | 29.8 | 26.0 | 28.4 | 35.0 | 35.0 |
| HS2 | 1 | 1 | 23.1 | 30.0 | 26.6 | 26.7 | 35.0 | 35.0 |
| HS2 | 1 | 1 | 23.2 | 31.1 | 27.2 | 27.3 | 35.0 | 35.0 |

| Cultivar | Inoculated micro-biome | Hours post inoculation of RLN | Subject ID | C_t_ *UBQ* | C_t_ *PR1* | C_t_ *PR17B* | C_t_ *PRX7* | C_t_ *HSP70* | C_t_ *GSL6* | C_t_ *CSD1* |
| --- | --- | --- | --- | --- | --- | --- | --- | --- | --- | --- |
| Beysehir | 1 | 24 | 1 | 20.1 | 23.9 | 26.1 | 26.9 | 20.9 | 23.3 | 17.2 |
| Beysehir | 1 | 24 | 1 | 20.0 | 24.7 | 25.9 | 26.8 | 21.4 | 22.1 | 17.6 |
| Beysehir | 1 | 24 | 2 | 18.6 | 24.9 | 27.0 | 24.5 | 18.9 | 21.6 | 16.9 |
| Beysehir | 1 | 24 | 2 | 18.6 | 25.7 | 27.5 | 24.7 | 19.9 | 21.5 | 17.1 |
| Beysehir | 1 | 24 | 3 | 23.0 | 27.9 | 30.2 | 28.0 | 26.0 | 26.9 | 21.8 |
| Beysehir | 1 | 24 | 3 | 23.1 | 28.1 | 30.7 | 28.5 | 27.1 | 26.6 | 21.7 |
| Beysehir | 1 | 24 | 4 | 20.1 | 23.9 | 26.1 | 26.4 | 22.1 | 24.3 | 18.6 |
| Beysehir | 1 | 24 | 4 | 20.5 | 25.0 | 27.1 | 26.5 | 22.8 | 24.6 | 18.9 |
| Beysehir | 1 | 24 | 5 | 23.5 | 29.2 | 29.8 | 28.7 | 24.4 | 25.1 | 20.4 |
| Beysehir | 1 | 24 | 5 | 23.6 | 30.3 | 30.8 | 29.1 | 25.3 | 24.9 | 20.3 |
| Beysehir | 1 | 24 | 6 | 20.6 | 22.5 | 26.2 | 24.6 | 19.1 | 21.8 | 16.7 |
| Beysehir | 1 | 24 | 6 | 20.2 | 23.4 | 25.9 | 24.5 | 20.0 | 21.6 | 17.1 |
| Beysehir | 1 | 72 | 7 | 21.5 | 25.1 | 25.3 | 28.1 | 23.6 | 22.1 | 17.0 |
| Beysehir | 1 | 72 | 7 | 21.6 | 25.6 | 25.6 | 28.3 | 24.1 | 21.4 | 17.2 |
| Beysehir | 1 | 72 | 8 | 20.3 | 23.0 | 25.2 | 27.2 | 19.6 | 21.6 | 17.2 |
| Beysehir | 1 | 72 | 8 | 20.0 | 23.9 | 25.4 | 27.3 | 20.6 | 21.2 | 17.1 |
| Beysehir | 1 | 72 | 9 | 20.6 | 24.0 | 24.9 | 26.9 | 20.8 | 21.5 | 16.6 |
| Beysehir | 1 | 72 | 9 | 20.8 | 24.5 | 24.9 | 27.3 | 21.5 | 21.1 | 16.6 |
| Beysehir | 1 | 72 | 10 | 20.2 | 22.9 | 23.7 | 27.1 | 21.1 | 20.5 | 16.3 |
| Beysehir | 1 | 72 | 10 | 20.5 | 23.4 | 24.5 | 27.2 | 22.1 | 20.4 | 16.6 |
| Beysehir | 1 | 72 | 11 | 21.1 | 22.5 | 26.1 | 28.4 | 22.7 | 22.4 | 17.6 |
| Beysehir | 1 | 72 | 11 | 20.9 | 23.9 | 25.5 | 28.6 | 23.5 | 22.5 | 18.1 |
| Beysehir | 1 | 72 | 12 | 23.0 | 27.2 | 26.9 | 29.9 | 23.4 | 23.1 | 18.2 |
| Beysehir | 1 | 72 | 12 | 23.5 | 27.9 | 27.2 | 29.6 | 24.3 | 22.4 | 18.4 |
| Beysehir | 0 | 24 | 13 | 20.4 | 27.0 | 26.9 | 27.2 | 22.3 | 23.2 | 19.0 |
| Beysehir | 0 | 24 | 13 | 20.1 | 26.5 | 29.3 | 27.1 | 23.0 | 22.7 | 18.3 |
| Beysehir | 0 | 24 | 14 | 20.5 | 25.5 | 26.0 | 26.0 | 22.9 | 22.8 | 17.3 |
| Beysehir | 0 | 24 | 14 | 20.0 | 26.2 | 26.4 | 26.0 | 23.8 | 22.5 | 17.5 |
| Beysehir | 0 | 24 | 15 | 21.0 | 24.5 | 26.5 | 27.2 | 22.5 | 23.6 | 18.0 |
| Beysehir | 0 | 24 | 15 | 20.5 | 24.4 | 26.1 | 27.1 | 23.1 | 23.3 | 18.5 |
| Beysehir | 0 | 24 | 16 | 21.5 | 26.7 | 28.2 | 28.2 | 24.1 | 23.5 | 19.1 |
| Beysehir | 0 | 24 | 16 | 21.6 | 27.6 | 28.5 | 28.4 | 24.4 | 23.7 | 19.2 |
| Beysehir | 0 | 24 | 17 | 21.1 | 27.1 | 29.0 | 28.7 | 24.0 | 25.0 | 20.4 |
| Beysehir | 0 | 24 | 17 | 23.7 | 28.0 | 29.1 | 29.0 | 24.4 | 25.3 | 20.7 |
| Beysehir | 0 | 24 | 18 | 14.5 | 24.9 | 26.7 | 28.0 | 22.2 | 23.7 | 19.1 |
| Beysehir | 0 | 24 | 18 | 17.4 | 25.4 | 26.7 | 28.1 | 22.8 | 24.0 | 19.1 |
| Beysehir | 0 | 72 | 19 | 24.5 | 28.9 | 29.1 | 32.0 | 23.4 | 23.7 | 19.6 |
| Beysehir | 0 | 72 | 19 | 24.5 | 29.8 | 29.1 | 31.9 | 24.5 | 23.6 | 19.8 |
| Beysehir | 0 | 72 | 20 | 21.8 | 26.3 | 27.4 | 29.7 | 22.2 | 23.8 | 18.9 |
| Beysehir | 0 | 72 | 20 | 21.8 | 27.0 | 27.6 | 30.0 | 23.0 | 23.3 | 19.1 |
| Beysehir | 0 | 72 | 21 | 22.5 | 26.6 | 26.9 | 29.7 | 21.5 | 22.0 | 17.5 |
| Beysehir | 0 | 72 | 21 | 21.5 | 26.8 | 27.4 | 29.2 | 22.6 | 21.7 | 17.5 |
| Beysehir | 0 | 72 | 22 | 20.1 | 29.4 | 27.1 | 30.2 | 23.8 | 23.0 | 18.4 |
| Beysehir | 0 | 72 | 22 | 21.5 | 29.3 | 28.0 | 30.3 | 25.0 | 23.3 | 18.6 |
| Beysehir | 0 | 72 | 23 | 23.7 | 27.8 | 27.9 | 30.9 | 24.0 | 23.2 | 19.1 |
| Beysehir | 0 | 72 | 23 | 22.3 | 28.2 | 28.3 | 30.6 | 24.9 | 23.4 | 19.4 |
| Beysehir | 0 | 72 | 24 | 23.5 | 29.4 | 29.6 | 29.5 | 24.3 | 23.9 | 19.5 |
| Beysehir | 0 | 72 | 24 | 20.0 | 29.5 | 30.8 | 29.1 | 24.9 | 23.9 | 20.1 |
| Valentina | 1 | 24 | 25 | 19.0 | 25.1 | 24.8 | 25.4 | 19.5 | 24.2 | 18.5 |
| Valentina | 1 | 24 | 25 | 18.8 | 25.2 | 25.1 | 29.1 | 20.1 | 24.4 | 17.4 |
| Valentina | 1 | 24 | 26 | 20.2 | 26.1 | 26.2 | 24.6 | 24.2 | 28.2 | 20.6 |
| Valentina | 1 | 24 | 26 | 20.6 | 25.9 | 26.8 | 25.7 | 24.7 | 27.3 | 20.1 |
| Valentina | 1 | 24 | 27 | 19.8 | 26.8 | 25.8 | 25.6 | 20.9 | 23.0 | 17.6 |
| Valentina | 1 | 24 | 27 | 20.1 | 28.2 | 26.1 | 25.3 | 21.3 | 23.1 | 17.7 |
| Valentina | 1 | 24 | 28 | 19.7 | 25.9 | 25.2 | 25.1 | 19.6 | 22.1 | 16.8 |
| Valentina | 1 | 24 | 28 | 23.4 | 26.1 | 25.4 | 24.8 | 20.0 | 22.0 | 16.7 |
| Valentina | 1 | 24 | 29 | 21.1 | 25.5 | 26.6 | 27.6 | 21.0 | 23.5 | 19.3 |
| Valentina | 1 | 24 | 29 | 22.8 | 25.8 | 26.4 | 27.7 | 21.4 | 23.6 | 19.0 |
| Valentina | 1 | 24 | 30 | 20.7 | 24.8 | 26.4 | 25.6 | 20.8 | 22.4 | 18.4 |
| Valentina | 1 | 24 | 30 | 20.9 | 25.6 | 26.7 | 25.8 | 21.3 | 22.8 | 18.4 |
| Valentina | 1 | 72 | 31 | 20.8 | 30.3 | 27.6 | 29.1 | 21.0 | 23.0 | 18.3 |
| Valentina | 1 | 72 | 31 | 20.8 | 27.0 | 27.5 | 29.0 | 21.3 | 22.6 | 18.4 |
| Valentina | 1 | 72 | 32 | 20.6 | 25.8 | 25.3 | 28.7 | 21.4 | 22.0 | 17.4 |
| Valentina | 1 | 72 | 32 | 20.9 | 26.1 | 25.3 | 28.6 | 22.1 | 21.8 | 17.2 |
| Valentina | 1 | 72 | 33 | 22.0 | 27.1 | 26.8 | 30.0 | 21.8 | 22.4 | 18.5 |
| Valentina | 1 | 72 | 33 | 22.1 | 27.3 | 26.6 | 29.8 | 22.6 | 22.4 | 18.1 |
| Valentina | 1 | 72 | 34 | 21.3 | 26.5 | 25.3 | 29.4 | 22.2 | 22.4 | 18.5 |
| Valentina | 1 | 72 | 34 | 21.5 | 27.1 | 25.9 | 29.2 | 22.5 | 25.1 | 18.5 |
| Valentina | 1 | 72 | 35 | 21.5 | 25.3 | 26.5 | 28.5 | 21.5 | 22.9 | 18.0 |
| Valentina | 1 | 72 | 35 | 21.7 | 26.3 | 27.0 | 28.7 | 21.9 | 22.3 | 17.9 |
| Valentina | 1 | 72 | 36 | 22.6 | 28.2 | 27.5 | 29.0 | 22.4 | 24.2 | 18.4 |
| Valentina | 1 | 72 | 36 | 22.6 | 28.8 | 27.3 | 29.3 | 22.6 | 22.9 | 18.6 |
| Valentina | 0 | 24 | 37 | 19.6 | 24.2 | 24.7 | 27.2 | 19.6 | 24.1 | 17.2 |
| Valentina | 0 | 24 | 37 | 19.5 | 25.3 | 25.9 | 27.2 | 20.2 | 22.0 | 17.2 |
| Valentina | 0 | 24 | 38 | 20.9 | 26.4 | 24.9 | 28.7 | 19.4 | 22.7 | 17.9 |
| Valentina | 0 | 24 | 38 | 21.0 | 27.4 | 25.2 | 28.8 | 20.5 | 22.8 | 17.8 |
| Valentina | 0 | 24 | 39 | 18.1 | 23.7 | 22.8 | 24.0 | 22.6 | 22.2 | 16.9 |
| Valentina | 0 | 24 | 39 | 18.9 | 24.7 | 23.6 | 24.0 | 23.7 | 22.4 | 17.3 |
| Valentina | 0 | 24 | 40 | 18.9 | 24.7 | 23.1 | 25.6 | 19.5 | 21.7 | 16.8 |
| Valentina | 0 | 24 | 40 | 18.9 | 24.1 | 23.8 | 25.9 | 19.9 | 21.6 | 16.9 |
| Valentina | 0 | 24 | 41 | 20.5 | 25.6 | 25.6 | 26.3 | 21.5 | 23.8 | 18.1 |
| Valentina | 0 | 24 | 41 | 20.8 | 25.8 | 26.1 | 26.3 | 22.2 | 23.1 | 18.0 |
| Valentina | 0 | 24 | 42 | 19.9 | 26.3 | 25.5 | 26.4 | 20.3 | 22.9 | 17.7 |
| Valentina | 0 | 24 | 42 | 19.9 | 26.5 | 25.4 | 26.5 | 21.0 | 23.1 | 17.8 |
| Valentina | 0 | 72 | 43 | 23.3 | 30.0 | 28.0 | 33.1 | 20.6 | 26.1 | 19.6 |
| Valentina | 0 | 72 | 43 | 23.6 | 28.3 | 26.5 | 32.1 | 21.8 | 25.8 | 19.5 |
| Valentina | 0 | 72 | 44 | 21.0 | 29.4 | 26.9 | 30.1 | 21.3 | 23.1 | 18.2 |
| Valentina | 0 | 72 | 44 | 22.7 | 27.8 | 27.2 | 30.1 | 22.2 | 26.0 | 18.5 |
| Valentina | 0 | 72 | 45 | 22.9 | 28.5 | 25.0 | 33.6 | 21.3 | 23.3 | 18.5 |
| Valentina | 0 | 72 | 45 | 22.8 | 26.7 | 25.4 | 31.9 | 22.2 | 23.5 | 18.4 |
| Valentina | 0 | 72 | 46 | 20.2 | 26.5 | 24.9 | 27.5 | 19.8 | 22.3 | 17.7 |
| Valentina | 0 | 72 | 46 | 20.2 | 25.7 | 25.1 | 29.0 | 20.4 | 22.2 | 17.6 |
| Valentina | 0 | 72 | 47 | 21.8 | 27.3 | 25.0 | 29.4 | 21.6 | 23.1 | 17.7 |
| Valentina | 0 | 72 | 47 | 20.7 | 27.5 | 25.1 | 29.2 | 21.8 | 23.3 | 18.1 |
| Valentina | 0 | 72 | 48 | 24.0 | 29.9 | 27.4 | 32.1 | 24.1 | 23.9 | 19.6 |
| Valentina | 0 | 72 | 48 | 24.7 | 30.1 | 27.0 | 32.6 | 24.9 | 23.8 | 19.6 |

**Supplementary Data S5.** Statistical analyses by procedure GLIMMIX (SAS 9.4)

*1. Responsiveness of modern cultivars and ancestral genotypes of barley to inoculated microbiome regarding plant growth and invasion of root-lesion nematodes*

Dependent variables (dv): Shoot weight (sfw), root weight (rfw), log-transformed counts of root-lesion nematodes in root (logRLN). Log-transformation resulted in a better model fit (AIC=22 compared to AIC=2472 without transformation), qq-Plot was much better with normal than Poisson distribution of log-transformed data.

Fixed effects: microbiome (inoculated, not), elite_cultivar (elite, ancestral)

Random effects: genotype (HB028, HB031, ..., Yusuf), experiment (JKI, TMU)

PROC GLIMMIX DATA=microbiome_effect PLOTS=all;

CLASS microbiome elite_cultivar genotype experiment;

MODEL dv = microbiome elite_cultivar microbiome*elite_cultivar / DDFM=kr;

RANDOM genotype experiment;

run;


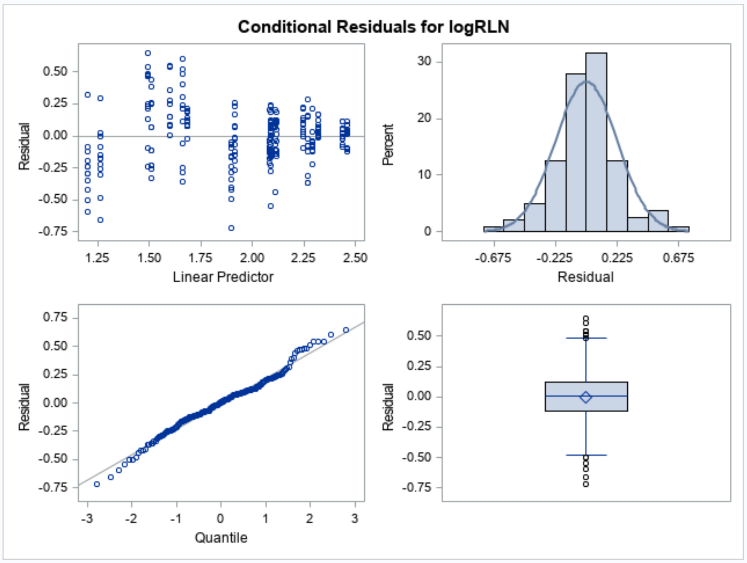

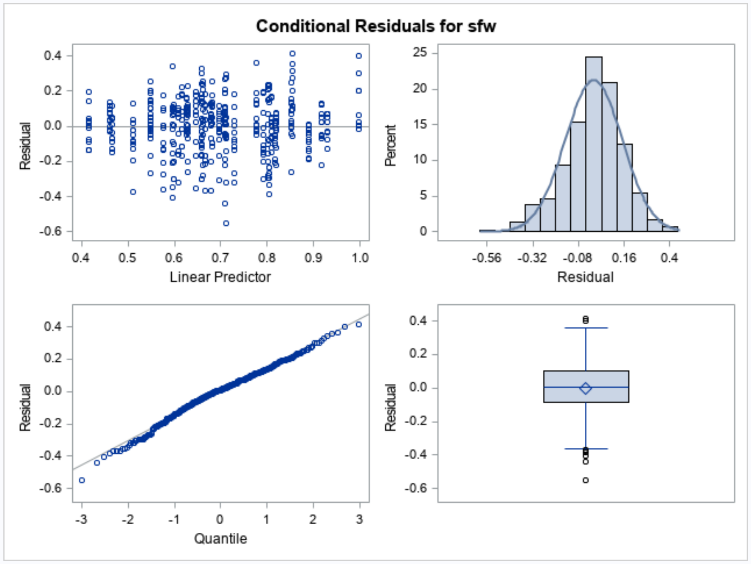


*2. Microbial density in the rhizosphere of elite cultivars and ancestral genotypes of barley, 20 days after inoculation of soil microbiome to the roots*

Dependent variable: log-transformed counts of colony forming units (logCFU)

Fixed effects: elite_cultivar (elite, ancestral), incubation time of R2A plates (1, 2, 7 days)

Random effects: genotype (HB028, HB031, ..., Yusuf), experiment (JKI, TMU)

Random: Repeated measures of CFU counts from the same rhizosphere (subject)

title "GLMM on elite cultivar vs. ancestral genotype";

PROC GLIMMIX DATA=cfu PLOTS=all;

CLASS elite_cultivar inctime genotype experiment;

MODEL logCFU = elite_cultivar inctime elite_cultivar*inctime / DDFM=kr;

RANDOM genotype experiment;

RANDOM _residual_ / SUBJECT=subjectID; /* repeated measures of CFU counts*/

run;

title "Post-hoc Tukey test on genotype";

PROC GLIMMIX DATA=cfu PLOTS=all;

CLASS inctime genotype experiment;

MODEL logcfu = elite_cultivar inctime elite_cultivar*inctime / DDFM=kr;

RANDOM experiment;

RANDOM _residual_ / SUBJECT=subjectID;

LSMEANS genotype / LINES ADJUST=tukey ADJDFE=row PLOTS=meanplot CL;

run;

*3. Microbiome effect on defense gene expression (-ΔΔC_t_) of the cultivars Beysehir and Valentina*

PROC GLM DATA=gene_expression PLOTS=all;

CLASS genotype dpi;

MODEL PR1 PR17B PRX7 HSP70 GSL6 CSD1 = genotype | dpi;

MANOVA h=_all_ / printe printh;

run;

*4. Microbiome effect on defense gene response to root-lesion nematode infestation in roots of modern cultivars (Jolgeh, Yusuf) and ancestral genotypes (HS1, HS2) of barley*

Dependent variables: gene1 - gene5: *EXPB1, PR1, PR5, LOX2, AOS*

PROC GLIMMIX DATA= gene_expression2 PLOTS=all;

CLASS microbiome genotype elite_cultivar;

MODEL gene = microbiome | elite_cultivar / DDFM=kr; /* gene: gene1 - gene5 */

RANDOM genotype;

run;

title "MANOVA";

PROC GLM DATA=microbiome_effect PLOTS=all;

CLASS microbiome elite_cultivar genotype;

MODEL gene1 - gene5 = microbiome | elite_cultivar;

MANOVA h=_all_ / printe printh;

run;

**Supplementary Figure S1.** Responsiveness of elite cultivars and ancestral genotypes of barley to rhizosphere microbiomes regarding invasion of root-lesion nematodes (RLN, *Pratylenchus neglectus*) within two weeks. Mean log-transformed numbers of RLN per gram of root from two independent experiments are separated on the horizontal axis (Experiment 1: ancestral genotypes HB047, HB049, and HB050, vs. elite cultivars Beysehir, Igri, and Valentina; Experiment 2: ancestral genotypes HS1 and HS2 vs. elite cultivars Jolgeh and Yusuf). Error bars indicate SE (n=12 plants with (+) and 12 plants without (-) inoculated microbiome per genotype of barley).


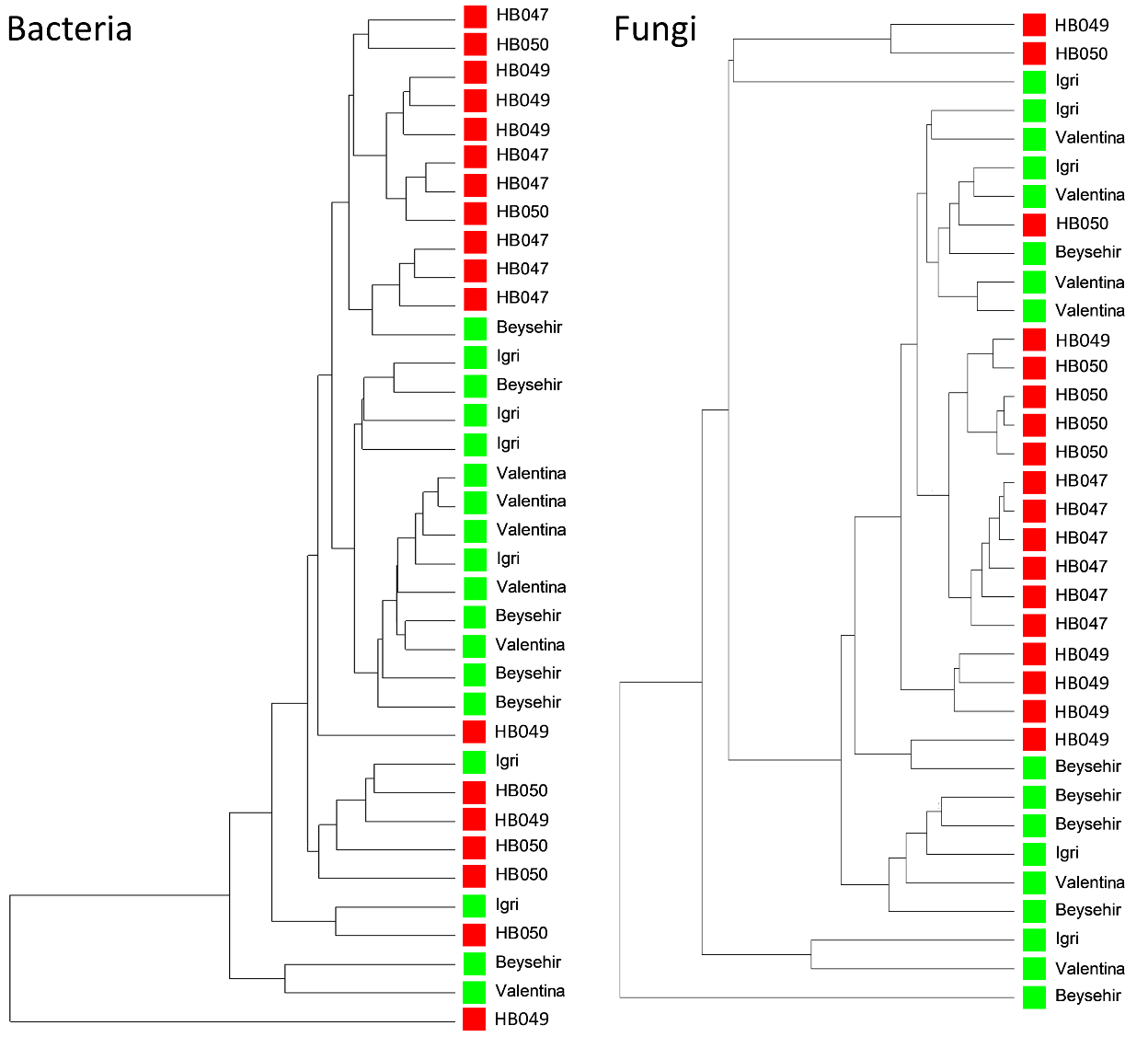


**Supplementary Figure S2.** Dendrograms indicating the similarity of bacterial or fungal communities in the rhizosphere of modern cultivars (green) and ancestral genotypes (red) of barley, based on the similarity matrices of community profiles by denaturing gradient gelelectrophoresis (Fig. 4 and 5, respectively). Clusters based on pairwise Pearson correlation of community profiles were constructed by UPGMA (Unweighted Pair Group Method with Arithmetic Mean) in GelCompar II 6.6 (Applied Maths, Sint-Martens-Latem, Belgium).
